# Oxidative stress response biomarkers in gills and liver of bullfrog tadpoles semi-chronically exposed to combined microplastics and titanium dioxide nanoparticles

**DOI:** 10.1007/s10646-026-03110-y

**Published:** 2026-07-02

**Authors:** Gabriel Hiroshi Fujiwara, Raquel Fernanda Salla, Thiago Lopes Rocha, Camila Sena dos Santos, Cleoni dos Santos Carvalho

**Affiliations:** 1 Postgraduate Program in Biotechnology and Environmental Monitoring (PPGBMA), Federal University of São Carlos, Sorocaba Campus, João Leme dos Santos Highway, Km 110, ZIP code, Sorocaba, São Paulo, SP-264, 18052-780 Brazil; 2 Institute of Tropical Pathology and Public Health, Federal University of Goiás, Goiânia, Brazil; 3 Department of Biology, Federal University of São Carlos, João Leme dos Santos Highway, 110 km, Postal Code, Sorocaba, São Paulo, 18052-780 Brazil

**Keywords:** Amphibians, Microplastics, Biomarkers, Plastic pollution, Nanomaterials, Emerging contaminants

## Abstract

Amphibians are among the most threatened groups of vertebrates worldwide, and the increasing release of emerging contaminants such as microplastics (MPs) and nanoparticles (NPs) poses a growing ecological risk to these organisms. This study evaluated the semi-chronic effects of polyethylene microplastics (60 mg/L) (PE-MPs) and titanium dioxide nanoparticles (10 µg/L) (TiO_2_ NPs), individually and in combination (Mix), on oxidative stress and detoxification biomarkers in the gills and liver of bullfrog tadpoles (*Aquarana catesbeiana*, in Gosner stages 25–26). Tadpoles were exposed for 15 days, and biochemical parameters (ethoxyresorufin-O-deethylase – EROD, glutathione-s-transferase - GST, superoxide dismutase - SOD, catalase - CAT, lipid peroxidation - LPO, protein carbonyls - PCO, and glucose levels) were quantified. In the gills, the Mix group significantly increased SOD and CAT activities, indicating sustained antioxidant responses associated with the formation of reactive oxygen species (ROS). In the liver, GST activity was elevated only in the Mix group, while LPO levels increased in all contaminant-exposed groups, demonstrating oxidative damage to membranes. Conversely, hepatic glucose concentrations decreased exclusively in the MP group, suggesting disturbances in energy metabolism potentially linked to contaminant bioaccumulation. Principal Coordinates Analysis (PCoA) and Integrated Biomarker Response indices (IBRv2) revealed organ-specific and treatment-dependent effects, with more pronounced biochemical alterations in the liver and stronger integrated responses in the MPs and Mix treatments. Altogether, these results demonstrate that MPs and NPs induce distinct oxidative and metabolic dysfunctions in tadpoles, with synergistic effects emerging under combined exposure. These findings highlight the importance of assessing mixtures of emerging contaminants and underscore the vulnerability of amphibian larvae to environmentally realistic pollution scenarios.

**Supplementary Information:**

The online version contains supplementary material available at https://doi.org/10.1007/s10646-026-03110-y.

## Introduction

According to the Second Global Amphibian Assessment (Global Amphibian Assessment 2 – GAA2), approximately 41% of amphibian species are threatened with extinction, making them the most vulnerable vertebrate group (RE: wild et al., 2023). The principal pressures acting on these vertebrates include habitat loss and degradation (93%), climate change (29%), infectious diseases, particularly chytridiomycosis (22%), wildfires (21%), the introduction of invasive alien species (14%), and overexploitation for commercial purposes, including human consumption, traditional medicine, and the exotic pet trade (9%) (Re: wild et al., 2023).

GAA2 indicates that pollution affects approximately 30% of amphibian species through habitat loss. In addition to environmental degradation, pollution contributes to climate change, disrupts amphibian biological functions and increasing susceptibility to fungal pathogens such as those causing chytridiomycosis (Re: wild et al., 2023). Among emerging pollutants, MPs and NMs have received growing attention due to their physicochemical complexity and potential effects on amphibians (Jacintho et al. 2025).

MPs are emerging contaminants defined as solid synthetic polymers ranging from 1 μm to 5 mm that have gained increasing attention in public debates, global policy agendas, and scientific investigations.

A recent study compiling and analyzing data from 706 articles on the abundance of MPs in different water bodies worldwide, conducted by An et al. (2025), estimated that the average concentration of MPs in surface waters is approximately 1.81 × 10⁴ items/m³, considering a size threshold of 100 μm.

In amphibians, particularly tadpoles, laboratory studies have shown that MPs bioaccumulate in the gills, liver, and intestine, impair growth and increase susceptibility to chytridiomycosis (Rahman et al. 2024).

NMs constitute another contaminant group attracting increasing scientific attention. These compounds, typically ranging from 1 nm to 100 nm in size, are used as constituents of cosmetics, pharmaceuticals, and electronic devices. Azimzada et al. (2021), investigated the concentration of titanium nanoparticles (Ti-NPs), cerium nanoparticles (Ce-NPs), and silver nanoparticles (Ag-NPs) in surface waters from 13 countries across 46 sampling sites, and reported average concentrations of 10 µg/L for Ti-NPs, 100 ng/L for Ce-NPs, and 1 ng/L for Ag-NPs.

Like MPs, NPs can induce oxidative stress, tissue damage in the gills and liver, mutagenicity, and bioaccumulation in tadpoles (Jacintho et al. 2025). In addition, NPs can also interact with other pollutants, serving as carriers for other chemicals, and are capable of generating reactive oxygen species such as superoxide radicals and hydroxyl radicals through photocatalytic reactions triggered by UV or visible light, or even through the oxidation of proteins adsorbed onto their surface, forming a protein corona (Jacintho et al. 2025).

With the increasing global demand for plastics and NMs, the growing release of these materials into the environment may disrupt natural habitats of amphibians (Jacintho et al. 2025). Tadpoles are particularly vulnerable because they remain restricted to aquatic environments until metamorphosis (Salla et al., 2024). Exposed to pollution throughout development, could potentially result in stress responses, behavioral and developmental alterations, as well as molecular and tissue damage. Additionally, tadpole gills are specialized in performing gas exchange and the regulation of hydroelectrolytic balance during amphibian larval stages. This organ is composed of vascularized gill arches and tufts, whose organization increases the surface area in contact with water, enhancing gas diffusion across cell membrane. In addition to these structural adaptations, tadpoles perform branchial ventilation, in which movements of the buccal and pharyngeal cavities maintain a continuous flow of water over the gills, ensuring adequate oxygenation (Schlenk and De Almeida 2023). However, such high permeability of the gill tissue can also make them more vulnerable to contaminants. Thus, it is plausible that MPs and NPs could accumulate and interact with gill tissues, causing inflammatory responses, histopathological changes, increased mucus production, oxidative stress, and tissue degeneration (Araújo et al. 2020).

Currently, few studies have investigated the interaction between MPs and NPs in amphibians. Among them, Salla et al. (2024) reported that a mixture of PE-MPs (60 mg/L) and TiO₂ NPs (10 µg/L) reduced survival and hatching rates in bullfrog embryos. In contrast, Jacintho (2025) observed that exposure to the same mixture did not induce genotoxicity or immunotoxicity in bullfrog tadpoles. However, when tested individually, both PE-MPs and TiO₂ NPs induced erythrocytic nuclear abnormalities (ENAs).

These two studies are pioneering in investigating the ecotoxicological effects of MPs and NPs in anurans; nevertheless, important biochemical gaps remain to be further explored. In this context, the present study primarily aimed to evaluate antioxidant responses, oxidative stress, and detoxification mechanisms in bullfrog tadpoles, *Aquarana catesbeiana* , at Gosner stages 25–26, following subchronic exposure (15 days) to PE-MPs (60 mg/L) and TiO₂ NPs (10 µg/L), both individually and in combination. Based on previous studies, our hypothesis is that both forms of exposure induce ecotoxicological effects, particularly those involving antioxidant responses, with distinct patterns among the different treatments. Furthermore, these analyses are expected to complement previous studies and contribute to the understanding of the biochemical mechanisms involved in adaptive responses in amphibians, particularly anuran larvae, as well as to provide support for future studies involving habitat conservation in pollution scenarios and environmental risk assessments.

## Materials and methods

### Microplastics and nanoparticles

PE-MPs (CAS No. 9002-88-4) were commercially obtained from Sigma-Aldrich. The full characterization of these MPs can be found in Salla et al. (2024) and Jacintho et al. (2025). The mean particle size was 35.46 μm; with mean density = 0.94 g/mL The shape of the PE-MPs was widely heterogeneous with an irregular surface, which represents a more realistic scenario expected to be found under natural environmental conditions. TiO_2_ NPs (CAS No. 13463-67-7) were purchased from Sigma-Aldrich. The characterization of TiO₂ NPs used in this study were previously described by Mamboungou et al. (2022). TiO₂ NPs had a rounded shape with a mean diameter of 20.5 ± 4.2 nm. In addition, the characterization of hydrodynamic diameter, polydispersity index (PdI) and zeta potential of PE MPs and TiO₂ NPs individually and in a mixture in dechlorinated tap water was previously conducted by Jacintho et al. (2025).

### Animal model

The complete procedures for animal acquisition and maintenance were previously described by Jacintho et al. (2025). Briefly, tadpoles at stage 25 Gosner were obtained from the “Laranjeiras” breeding facility (Gameleira de Goiás, Goiás, Brazil; 16°20′30.3″S / 48°44′17.8″W) and transported to the Federal University of Goiás, where they were maintained in the Environmental Ecotoxicology Laboratory (LABAE) for a 7-day acclimation period to allow recovery from handling and transportation stress. During the acclimation, tadpoles were kept in regular dechlorinated tap water under controlled conditions: temperature = 24 ± 1 °C; water pH = 7 ± 0.5; conductivity = 68 ± 1 µS/cm; dissolved oxygen > 40% (measured with a Multi-parameter Water Meter DR-500, Dongrun Co., Ltd, China); ammonia levels < 1 mg/L (Sera Ammonia Test, Sera GmbH, Germany); absence of chlorine (Sera Cl Test, Sera GmbH, Germany); hardness ≈ 3 dH (Sera GH Test, Sera GmbH, Germany); and a 12 h light / 12 h dark photoperiod, as recommended by standardized amphibian maintenance and research guidelines (Pough 2007).

### Experimental design

Tadpoles at 25–26 Gosner Stages were randomly assigned to four experimental groups: one control group (Control, *n* = 15), a second group exposed to 60 mg/L of PE-MPs (MP, *n* = 15), corresponding to approximately 4.24 × 10⁻⁶ particles/m³. A third group exposed to 10 µg/L of TiO₂ NPs (NP, *n* = 15); and a fourth group exposed to the combination of both contaminants (Mix, *n* = 15).

Each treatment was assembled in triplicate (totaling 45 tadpoles per experimental group), distributed into 20 L glass aquaria and maintained under a semi-static exposure system, with water renewal every 24 h (Araújo et al. 2020). The triplicates were composed of separate aquaria, with 3 aquaria containing 15 individuals each. The selected concentrations were based on previous studies employing environmentally relevant concentrations of MP and NP (individually) in amphibian ecotoxicology experiments (Salla et al. 2024; Jacintho et al. 2025). Therefore, the present study maintains an environmentally predictive approach and reflects realistic contamination scenarios.

Regarding the concentrations of MPs and NPs in the exposure solutions, we prepared new solutions prior to every water change by adding the particles to the water to reach the final concentration of 60 mg/L of microplastics, and 10 µg/L of TiO2 NPs. Each aquaria had a total final volume of 15 L of solution. Since neither MPs nor NPs are volatile, the concentration should only change if the animals absorbed or ingested the particles during the exposure days, which was part of the expected toxicological routes for this experiment. Therefore, it was already expected that the concentration of the toxicants in the water could vary between every water change, depending on how much of the toxicants the tadpoles would daily absorb and/or re-eliminate (through excretion) along the whole exposure period. We also added this information to the methods to improve the methods.

Throughout the 15-day exposure period, animals were maintained under the same dechlorinated tap water and controlled physico-chemical conditions, such as dissolved oxygen > 40% (measured with a Multi-parameter Water Meter DR-500), temperature (24 ± 1 ◦C), pH (7.2 ± 0.2), ammonia (0.018 ± 0.002 ppm), water hardness (3.0 ± 0.5 ◦dH) and chlorine (< 0.01 ppm). Tadpoles were fed once daily with commercial fish feed (45% protein, 14% extracts, 5% fiber, 14% minerals, and 87% dry matter). Mortality and the appearance of morphological or behavioral abnormalities were monitored and recorded daily. After 15 days of exposure there was no mortality in response to any of the experimental groups, and tadpoles were euthanized by cranial concussion, following the guidelines of the (American Veterinary Medical Association 2020). The organs were collected and immediately frozen at − 80 °C, and subsequently transported to the Biomarkers Laboratory (LaBioM), UFSCar, Sorocaba campus, with the samples stored in Eppendorf tubes inside an insulated container with dry ice.

### Biochemical biomarkers

A total of 24 gill samples (*n* = 6 per experimental group) and 24 liver samples (*n* = 6 per experimental group) were used for biochemical analyses.

During tissue excision, only the right gill lamellae and the right hepatic lobe were dissected for these assays. All sampled tissues appeared morphologically similar and consistent with the normal anatomical characteristics described in the literature. Each tissue sample was individually homogenized using an ULTRA-TURRAX^®^ tissue homogenizer (IKA^®^ T10 basic) in two different volumes of phosphate-buffered saline (PBS): 800 µL for liver samples and 400 µL for gill samples. The buffer solution consisted of 1.365 M NaCl, 0.027 M KCl, 0.054 M Na₂HPO₄·7 H₂O, and 0.018 M KH₂PO₄, adjusted to pH 7.2 and kept at 4 °C. The homogenate was centrifuged at 10,000 g (HERMLE Z 323 K) for 30 min at 4 °C. Aliquots of the supernatant were used to determine total protein levels according to Bradford (1976), using bovine serum albumin as the standard and absorbance readings at 595 nm in microplates. All biochemical analyses were performed in duplicates using a BioTek Synergy HTX microplate reader.

The activity of EROD was determined according to Kennedy and Jones (1994). Briefly, the assay is based on the ability of the cytochrome P450 system, particularly the CYP1A enzyme, to convert ethoxyresorufin into resorufin, which is quantified by fluorescence using an excitation filter at 530 nm and an emission filter at 590 nm, with enzyme activity being monitored for 30 min at 30-second intervals. GST activity was measured following the method of Keen et al. (1976), in which GST activity is assessed based on its ability to catalyze the reaction between the substrate 1-chloro-2,4-dinitrobenzene (CDNB) and reduced glutathione (GSH), forming a thioether that can be monitored kinetically by the increase in absorbance at 340 nm, for 5 min at 30-second intervals.

SOD and CAT activities were determined according to Flohe (1984) and Aebi (1984), respectively. Briefly, SOD activity was calculated by monitoring its ability to inhibit the rate of cytochrome c reduction, measured at 550 nm, for 10 min at 5-second intervals, using the xanthine–xanthine oxidase system as a source of ROS. CAT activity was assessed based on its capacity to catalyze the decomposition of H₂O₂ into water and was quantified by the decrease in absorbance at 240 nm, for 10 min at 5-second intervals.

Analyses of the LPO biomarker and the PCO were performed according to Buege and Aust (1978) and Colombo et al. (2016), respectively. Briefly, LPO was quantified based on the content of thiobarbituric acid reactive substances (TBARS), formed by the breakdown of polyunsaturated fatty acids because of ROS, which react with thiobarbituric acid to produce compounds with an absorbance peak at 535 nm. PCO concentrations were determined from the reaction between carbonylated proteins and 2,4-dinitrophenylhydrazine (DNPH), producing hydrazone derivatives that were monitored at 370 nm.

Glucose concentration was determined using the Labtest Liquiform kit No. 133. The concentration of this metabolite was normalized to the protein content of the homogenate and expressed in µmol/mg of protein. Its determination was based on the equation obtained from the calibration factor of the kit standard (100 mg/dL) at 505 nm. In this study, all seven biomarkers were evaluated in liver samples. For gill samples, due to the low tissue volume per sample, only GST, SOD, CAT, and PCO were analyzed.

Additionally, the ratio of protein content (mg) to frozen tissue mass (mg) was calculated for the gill samples. In this study, only the weighing of the gills was performed, since the liver analyses were performed directly. To ensure sample integrity, empty Eppendorf tubes were previously sterilized with 70% ethanol and stored at − 80 °C for 24 h to match the temperature conditions of the biological samples. The frozen tissues were removed from the ultra-freezer, kept on ice, and carefully transferred with fine stainless steel tweezers to pre-chilled and pre-weighed Eppendorf tubes, placed on a styrofoam rack to minimize temperature variation. The tubes were immediately sealed and weighed on an analytical balance to determine the tissue mass. Then, chilled buffer (4 °C) was added, and the tissues were promptly homogenized to prevent enzymatic degradation. To ensure analytical consistency and minimize enzymatic degradation, all enzyme biomarker assays and total protein quantifications were performed on the same day. The PCO content was measured the following day using aliquots stored at − 80 °C until analysis.

### Statistics

The sample size chosen for the biochemical analysis (*n* = 6 per each experimental group) was determined using the software GPower (version 3.1) and applying the “a priori” function to estimate a good sample size for each of our biomarkers. The analysis was based on the effect sizes reported in previous literature assessing biochemical responses of amphibians to toxicants, together with a significance level (α) of 0.05 and a statistical power (1 − β) of 0.80. Under these assumptions, the analysis indicated that a minimum sample size of six tadpoles per experimental group would be sufficient to detect toxicologically relevant responses, as well as to adhere to ethical principles aimed at reducing animal use in experimental research.

Outliers within each experimental group were identified using Dixon’s Q test, with p-values < 0.05 considered significant, and the modified Z-score based on the Median Absolute Deviation (MAD), where Z-scores > 3.5 were classified as outliers. Winsorization was applied to correct data points identified as outliers. Residual normality for each analysis was assessed using the Shapiro-Wilk test, with normality accepted for *p* ≥ 0.05, complemented by visual inspection of Q-Q plots. Homoscedasticity of the experimental data was evaluated using Levene, Brown-Forsythe, Fligner-Killeen, Breusch-Pagan, and Goldfeld-Quandt tests, with *p* ≥ 0.05 indicating homoscedasticity.

Influential points were assessed using Cook’s distance, with values above 0.902 considered influential. The threshold was calculated as described by Chatterjee and Hadi (2015), where points above 50% of the F-distribution [F(*p* + 1, n–p–1)]—with p representing the number of experimental groups (*p* = 4) and n the total number of observations (*n* = 24)—may be considered influential for ANOVA analyses. In this study, all analyses followed the F-distribution F(5, 19). Box-Cox transformation was applied only when a dataset violated the assumptions of normality and homoscedasticity. Transformed data were used for group comparisons; however, for clarity in interpretation, figures were constructed from the original, untransformed data.

Omnibus tests including ANOVA, Welch ANOVA, and Kruskal-Wallis were employed according to the fulfillment of their respective assumptions, with p-values < 0.05 considered statistically significant. Post-hoc analyses included Tukey HSD (Honest Significant Difference), Games-Howell, and Dunn tests, with p-values adjusted using the Bonferroni method. Effect sizes were also calculated as partial η² (ANOVA), ω² (Welch ANOVA), and η²_H_ (Kruskal-Wallis) (Morgan 2017).

A multivariate Principal Coordinates Analysis (PCoA) was performed, with envfit vectors (available in the R package “vegan”) used to fit independent variables onto the ordination plot. To assess the overall variation of experimental factors, a PERMANOVA test with 10,000 permutations was conducted, with p-values < 0.05 considered significant. This information is provided in the Supplementary Material.

IBRv2 indices (Sanchez et al. 2013) were calculated, and radar plots were generated to evaluate the behavior and effects of different biomarkers in response to microplastic and nanoparticle exposures.

All statistical analyses were performed using R software, version 4.3.3, with the packages “dplyr”, “IBRtools”, “tidyr”, “tibble”, “forcats”, “pastecs”, “outliers”, “lawstat”, “lmtest”, “rstatix”, “coin”, “WRS2”, “RVAideMemoire”, “permuco”, “MASS”, “DescTools”, “robustHD”, “ggplot2”, “ggsignif”, “ggpubr”, “ggtext”, “gridExtra”, and “vegan”.

## Results and discussion

### Biomarker responses in tadpole gills

**Fig. 1:**
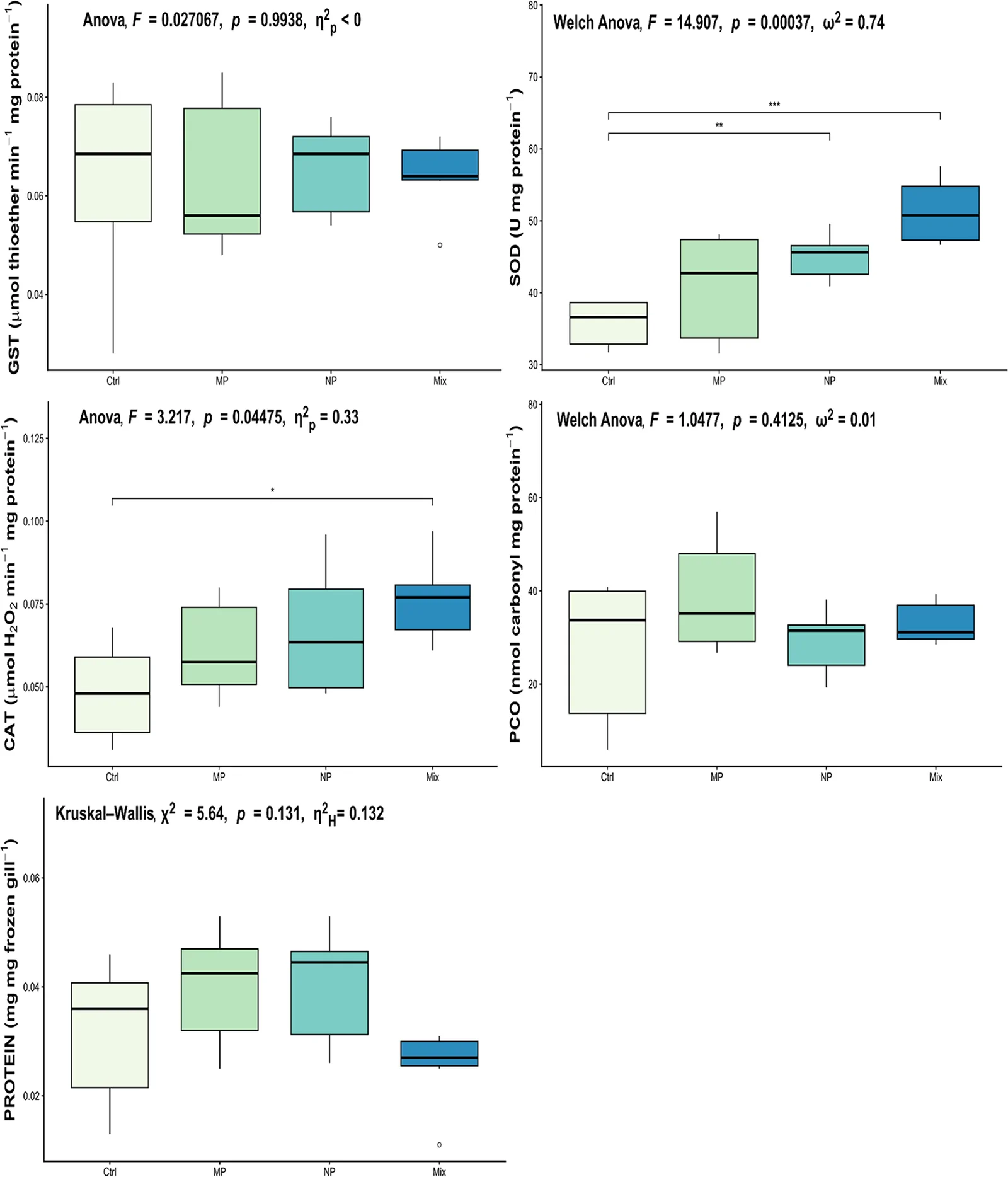
Biomarker responses in the gills of *Aquarana catesbeiana* tadpoles in the control group (Ctrl) and those exposed to polyethylene microplastics (60 mg/L, MP), titanium dioxide nanoparticles (10 µg/L, NP), either individually or combined (Mix), after a 15-day chronic exposure. The top of each graph shows the statistical values for each test (F and χ²), their respective p-values, and the corresponding effect sizes (η²p, η²H, and ω²). Significance levels are indicated with asterisks: *, *p* < 0.05; **, *p* < 0.01; ***, *p* < 0.001

The biomarker responses for the four treatments are shown in Fig. 1. GST did not show significant alterations in any of the treatments. GSTs are part of a multifunctional enzyme superfamily that plays key roles in Phase II detoxification. They can catalyze conjugation reactions between GSH and endogenous or exogenous electrophilic agents, facilitating their subsequent elimination via multidrug resistance (MDR) proteins. Additionally, GSTs can catalyze the reduction of reactive and oxidizing species using GSH, which is converted into its oxidized form (GSSG) (Keen et al. 1976).

In our study, since these enzymes were able to catalyze detoxification reactions efficiently, after 15 days of exposure, the tadpoles likely achieved some level of acclimation to the environment, or an adaptive response to the experimental conditions themselves, in a manner comparable to the adjustments that occur in response to environmental variations, reducing the need for elevated enzymatic responses. However, further studies, both acute and chronic, are still necessary to confirm this hypothesis and to elucidate GST behavior under shorter exposure periods.

Significant increases in SOD activity were observed in the Mix (*p* = 0.0004) and NP (*p* = 0.003) groups compared to the control (Fig. 1). SOD plays a critical role in cellular detoxification, particularly in antioxidant reactions mediated by ROS (Subaramaniyam et al. 2023). It catalyzes the dismutation of two superoxide anions into one molecule of H₂O₂ and one of molecular oxygen. The results of this study indicate that even after 15 days of exposure, the interaction between TiO₂ NPs, either isolated or combined with MPs, continued to elicit detectable biochemical responses, suggesting that the organisms were still responding to environmental conditions.

Due to the high permeability and vascularization of gills, MPs combined with TiO₂ NPs and isolated NPs may have bioaccumulated in this organ, increasing ROS production either through interactions between the two compounds in intra- and extracellular compartments or via desorption of NPs from MP surfaces, leading to prolonged mobilization of the antioxidant system (Rozman et al. 2023). Additionally, the Mix group exhibited increased CAT activity (*p* = 0.0306) compared to the control, which indicates that the combination of these contaminants led to elevated cellular H₂O₂, potentially resulting from increased toxicity due to the combined substances or as a delayed enzymatic response caused by TiO₂ NP desorption. Typically, increases in SOD activity directly trigger CAT activity, as SOD generates H₂O₂ during ROS dismutation, although other biochemical processes, such as NADPH oxidase, xanthine oxidoreductase activity, or mitochondrial electron leakage, can also produce H₂O₂ (Subaramaniyam et al. 2023).

In this context, the CAT increase in the Mix group may be directly related to elevated SOD activity, but it may also reflect perturbations in other biochemical systems caused by the contaminants. Conversely, the lack of convergence between CAT and SOD activity in the TiO₂ NP group may be due to H₂O₂ being utilized by other antioxidant systems, such as glutathione peroxidase - GPx (Subaramaniyam et al. 2023).

No changes in PCO levels or protein content were observed (Fig. 1) and protein-to-frozen-tissue-mass ratio did not differ between treatments, indicating that contaminant exposure, whether isolated or combined, did not affect protein synthesis or structure.

Regarding nanoparticles, recent studies show that these materials can also bioaccumulate in tadpoles and cause various types of damage. Murthy et al. (2024) reported that *Duttaphrynus melanostictus* tadpoles (stage 26) chronically exposed for 30 days to iron oxide nanoparticles (20–40 nm; 5, 10, 50 mg/L) bioaccumulated the particles in blood, liver, and kidney, with dose-dependent biochemical and cellular effects.

In our results, combined exposure to MPs and NPs led to significant increases in SOD and CAT activities in the gills, whereas isolated NP exposure increased only SOD activity. In contrast, PCO levels and the protein-to-tissue weight ratio did not show significant changes compared with the control. These findings may indicate a condition of sublethal oxidative stress, in which the gills activated antioxidant mechanisms without detectable structural oxidative damage to proteins. This pattern is consistent with the initial generation of ROS induced by MPs and NPs (Subaramaniyam et al. 2023). These contaminants can interact with cellular membranes and mitochondria, altering electron transport and promoting the formation of superoxide (O₂•⁻), which triggers the activation of redox-sensitive pathways responsible for regulating antioxidant defenses (Subaramaniyam et al. 2023). In this context, the increase in SOD observed, particularly in the group exposed only to NPs, suggests a first-line antioxidant response, since this enzyme catalyzes the conversion of superoxide into H₂O₂. The absence of a concomitant increase in CAT in this treatment indicates that hydrogen peroxide production may have remained within the basal cellular neutralization capacity, possibly being compensated by other antioxidant systems, such as GPx.

On the other hand, the simultaneous increase in SOD and CAT in the mixture group suggests a higher oxidative burden, possibly associated with interactions between MPs and NPs. MPs can adsorb NPs through physisorption processes and act as vectors, increasing their retention and bioavailability in the gills and prolonging cellular exposure, which may intensify ROS generation.This increase in superoxide production and, consequently, H₂O₂ levels would require coordinated activation of CAT to prevent the formation of highly reactive hydroxyl radicals, characterizing a more robust antioxidant response compared with isolated exposure. This explanation is supplemented with the experiments conducted by Rozman et al. (2023), in which PE-MPs showed the ability to adsorb TiO₂ NPs as aggregates on their surface and exhibited a low desorption rate, favoring the persistence of these contaminants.

Despite these enzymatic alterations, the absence of changes in PCO levels and in the protein-to-tissue weight ratio indicates that antioxidant mechanisms were effective in preventing oxidative damage to structural proteins. This pattern suggests that, although the contaminants induced oxidative stress, the antioxidant capacity of the gills was sufficient to maintain cellular integrity during the experimental period, characterizing a compensatory adaptive response.

### Biomarker responses in the liver of tadpoles

**Fig. 2:**
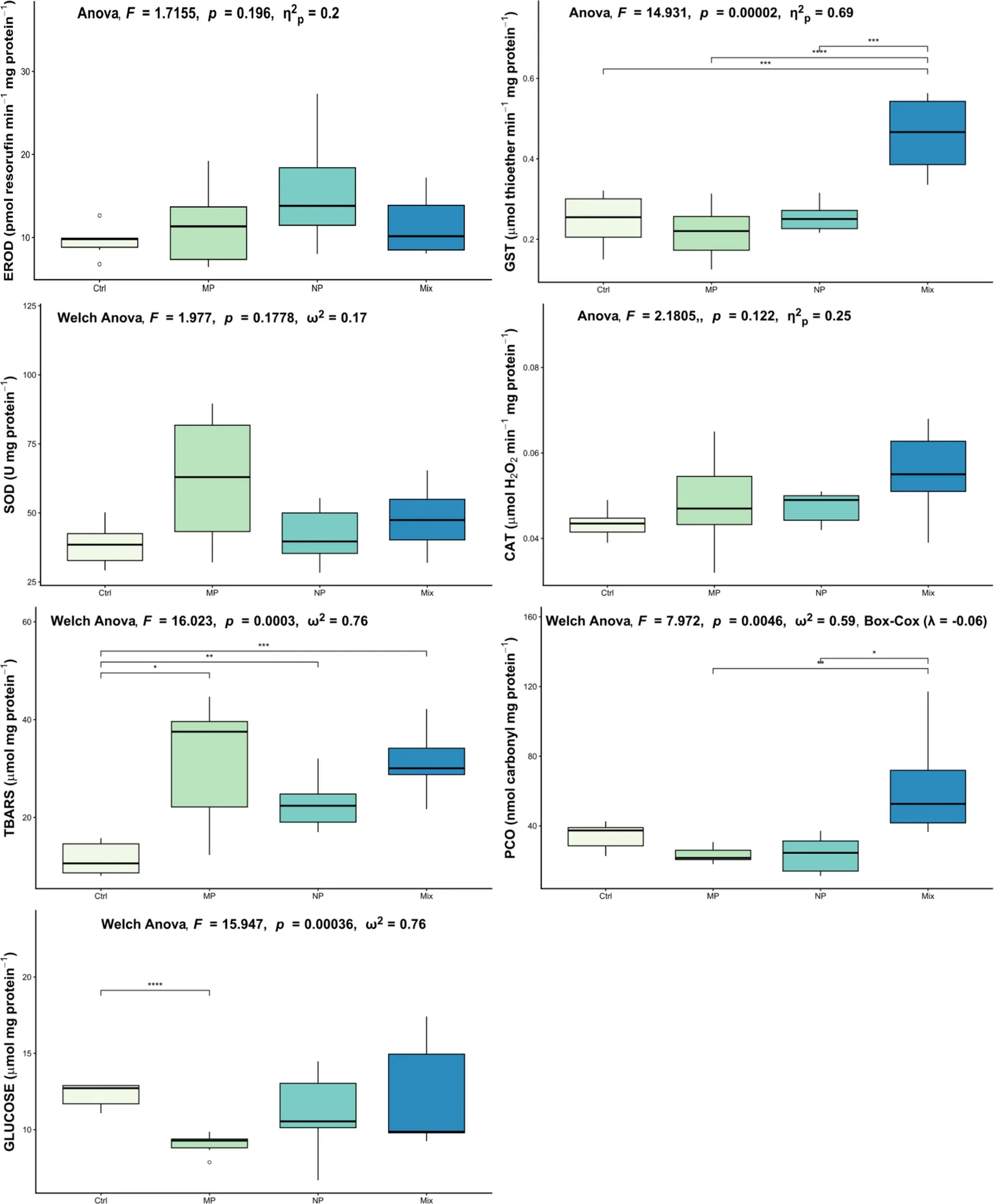
Biomarker responses in the livers of *Aquarana catesbeiana* tadpoles in the control group (Ctrl) and those exposed to polyethylene microplastics (60 mg/L, MP), titanium dioxide nanoparticles (10 µg/L, NP), either individually or combined (Mix), after a 15-day chronic exposure. The top of each graph shows the statistical values for each test (F), their respective p-values, and the corresponding effect sizes (η²p and ω²). PCO data were Box-Cox transformed, with the lambda value indicated on the graph. Significance levels are indicated with asterisks: *, *p* < 0.05; **, *p* < 0.01; ***, *p* < 0.001; ****, *p* < 0.0001

The EROD assay allows the evaluation of CYP1A1 activity, one of the main enzymes of the cytochrome P450 system involved in phase I detoxification, during which xenobiotics are converted into more electrophilic forms that are subsequently metabolized during phase II (Schlenk and De Almeida 2023). No significant differences in EROD activity were observed among the experimental groups (Fig. 2).

However, Yin et al. (2024) conducted in vitro assays using liver homogenates from juvenile *O. mossambicus* exposed to different concentrations of PS-MPs (1–100,000 µg/L) of varying sizes (0.1–10 μm). After 48 h of exposure, no differences in EROD activity were observed at any concentration; however, the combination of PS-MPs (100 µg/L) with other elements (Cu²⁺, Pb²⁺, Zn²⁺, flame retardants, and pharmaceuticals) at various concentrations led to a decrease in enzyme activity. Although MPs and NPs did not induce hepatic biochemical effects in our study, research in fish models indicates that the response of this enzyme may be sensitive to the interaction of MPs with other contaminants. We emphasize that further studies are needed to assess whether this sensitivity observed in fish also occurs in amphibians, as our results did not detect significant changes, which may indicate a resistance or adaptive response of the species to microplastics and nanoparticles following subchronic exposure.

The Mix group showed significantly higher GST activity compared to the Ctrl (*p* = 0.0002), MP (*p* = 0.00004), and NP (*p* = 0.0003) groups. The synergy between MPs and NPs can intensify cellular stress through the bioaccumulation of these contaminants in tissues and increased ROS generation. Even when adsorbed to the surface of MPs, NPs can preserve their photocatalytic activity, although this effect occurs in a more localized manner (Kalčíková et al. 2023). Furthermore, despite showing affinity for the PE-MP surface, these nanoparticles still retain desorption capacity (Rozman et al. 2023), and ingestion and absorption processes within the organism also need to be considered. This phenomenon can prolong cellular exposure and increase ROS production, promoting oxidative stress and, consequently, the activation of antioxidant defense systems. Under these conditions, the increased GST activity observed in tadpoles may be related to the greater production of ROS resulting from bioaccumulation and interactions between MPs and NPs in tissues, reflecting an adaptive response of the organism to neutralize reactive metabolites and limit oxidative damage.

LPO levels, measured via TBARS, were significantly higher in the MP (*p* = 0.047), NP (*p* = 0.01), and Mix (*p* = 0.001) groups compared to the Ctrl. Conversely, PCO concentrations in the Mix treatment were higher than in the MP (*p* = 0.009) and NP (*p* = 0.023) groups, but not significantly different from the Ctrl (Fig. 2).

The difference observed between TBARS and PCO responses may indicate that oxidative damage to lipids occurred more uniformly than protein oxidation during exposure. LPO is recognized as a sensitive and early marker of oxidative stress, as membrane lipids are highly vulnerable to attack by ROS (Pizzimenti et al. 2013). In contrast, PCO generally reflects more severe oxidative stress conditions, often associated with inflammatory processes or tissue degeneration. In this context, exposure to MPs and NPs, both individually and in combination, likely generated sufficient ROS to trigger lipid damage, but not enough to promote widespread protein oxidation detectable as an increase in total PCO levels.

Furthermore, LPO generates reactive aldehydes, such as malondialdehyde, 4-hydroxynonenal, and acrolein, which are electrophilic and highly reactive, tending to interact with nucleophilic regions of proteins (Pizzimenti et al. 2013). These interactions result in localized oxidative modifications rather than uniform oxidation across the entire cellular proteome (Pizzimenti et al. 2013). This mechanism may explain why the Mix group showed higher PCO levels compared to isolated exposures, although without a significant difference relative to the Ctrl group, suggesting that these changes were localized and diffuse.

These aldehydes also induce cellular detoxification systems, such as aldehyde dehydrogenases, alcohol dehydrogenases, and GST, which may also have contributed to the increase in this biomarker in the Mix group (Pizzimenti et al. 2013). In addition, under moderate oxidative stress conditions, cells may activate defense mechanisms, in which PCO may also act as a cellular signaling mechanism, including antioxidant systems, degradation of damaged proteins, and reductive pathways such as the thioredoxin system, which help limit damage accumulation (Chienwichai et al. 2019).

Taken together, the results of this study suggest a scenario of moderate oxidative stress, in which measurable lipid damage occurred, but protein oxidation was partially controlled by cellular defense systems, indicating an adaptive response of the organism to contaminant exposure.

However, we cannot rule out the possibility that the differences observed between LPO and PCO responses may be influenced by sample size. Although the sample size used complies with ethical animal use protocols, and the statistical analyses employed are appropriate for small samples (Morgan 2017), the variability in PCO responses among individuals in the Mix group may have contributed to the observed significance. From this perspective, further analyses of this biomarker are needed to determine whether these biochemical responses reflect physiological regulatory processes or whether they result from heteroscedasticity within the Mix group in the present analysis.

Our results indicate that, after 15 days, the exposure to PE-MPs and TiO_2_ NPs, whether isolated or combined, was able to increase the levels of cellular LPO products. The lack of response previously reported for the antioxidant biomarkers such as SOD and CAT may suggest that the LPO process occurred over a period sufficient for the organisms to adapt, leading to a stabilization of enzymatic responses while LPO products remained present in the tissues.

Hepatic glucose content showed a significant decrease in the MP group (*p* = 0.0001) compared to the Ctrl group. The reduction in glucose levels may result from multiple factors, including decreased food intake due to competition with MPs, glycogen depletion caused by MPs accumulation in the tissue, physiological responses involving oxidative stress and inflammatory processes—which can increase ATP consumption and, consequently, molecular glucose consumption—as well as alterations in regulatory enzymes of glucose metabolism, such as phosphoenolpyruvate carboxykinase (PCK), glucose-6-phosphatase (G6PC), and AMP-activated protein kinase (AMPK) (Subaramaniyam et al. 2023).

Jacintho et al. (2025) reported that *A. catesbeiana* tadpoles exposed to PE-MPs under conditions like our ecotoxicological assay showed no induction of immune or genotoxic responses in intracardiac blood samples. Based on our results, where no significant enzymatic oxidative stress responses (SOD and CAT) were detected in the MP group, and considering literature data, the decrease in glucose levels in this group may be related to bioaccumulation processes and imbalances in glucose metabolism-regulating enzymes.

Considering the complexity of the responses reported in different studies and species, further investigations are essential to elucidate the mechanisms underlying these results. Variations in biological characteristics, exposure pathways, types and sizes of polymers and nanomaterials, and physicochemical interactions can modulate toxicity pathways, leading to divergent physiological, biochemical, and molecular responses. Therefore, a broader and more integrative research effort is needed to determine how these factors interact and contribute to the observed patterns of organismal susceptibility.

### PCoA and IBRv2

#### Gill

**Fig. 3:**
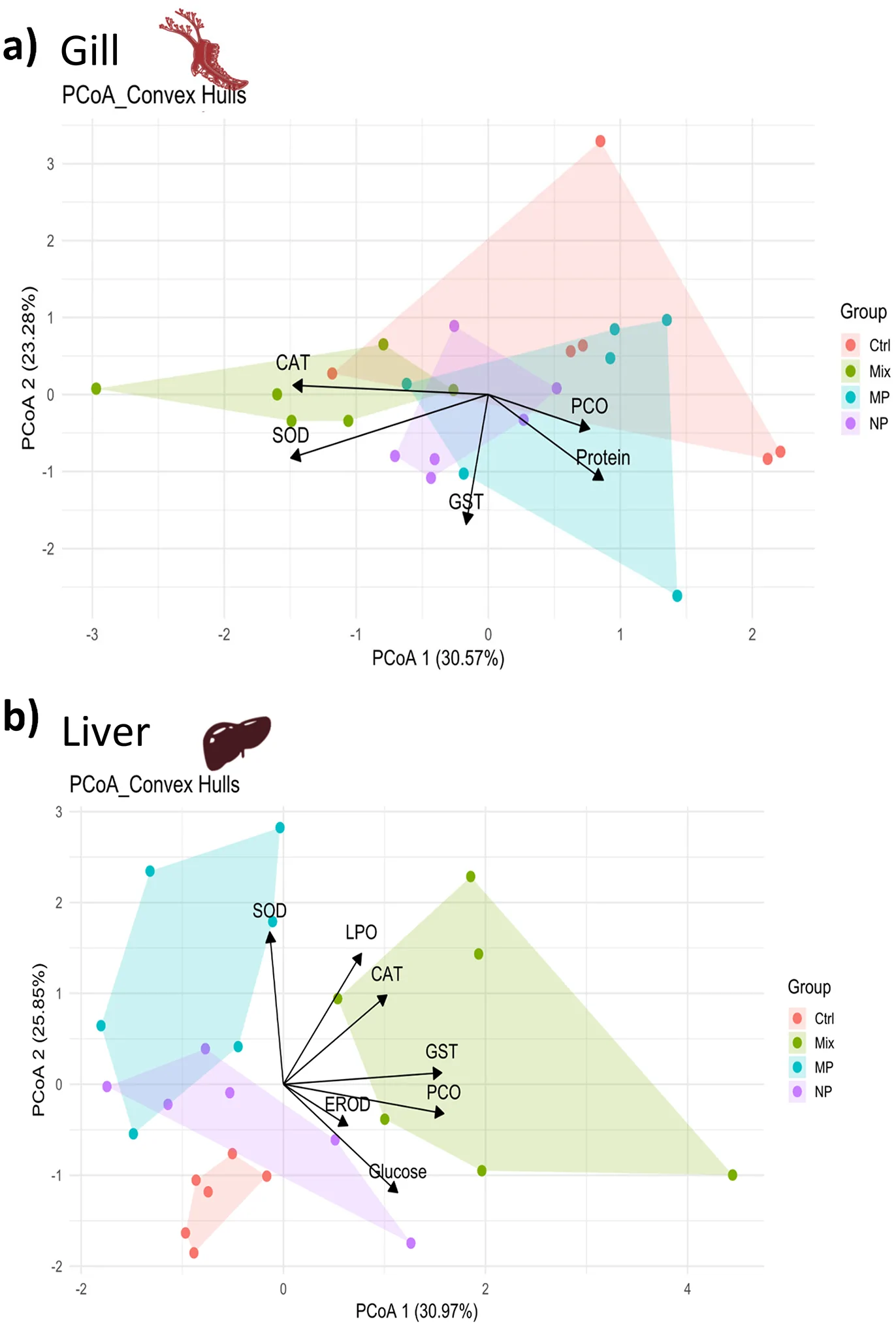
Principal Coordinates Analysis (PCoA) based on the Euclidean dissimilarity matrix of565 biochemical responses in Aquarana catesbeiana tadpoles exposed to different treatments: control (Ctrl),566 polyethylene microplastics (60 mg/L, MP), titanium dioxide nanoparticles (10 μg/L, NP), and the mixture567 (Mix). Colored regions represent each experimental group. The vectors correspond to the envfit adjustment568 of response variables, indicating the direction and magnitude of each biomarker’s contribution to the569 separation among groups. **a**) PCoA of tadpole gills; **b**) PCoA of tadpole liver

PERMANOVA revealed significant differences among experimental groups in the gills (*p* = 0.001). The first two axes explained 53.85% of the total variation (PCoA1 = 30.57%; PCoA2 = 23.28%) (Fig. 3a). Although group separation was statistically significant, spatial inspection indicated partial overlap among treatments.

The Mix group showed a convergency to separate along PCoA1, occupying negative axis scores. Vector fitting indicated that antioxidant enzymes (SOD and CAT) were the main contributors to the spatial segregation of this group. In contrast, PCO and protein levels were more strongly associated with the MP group, whereas the NP group was influenced by multiple variables without a single dominant contributor.

**Fig. 4:**
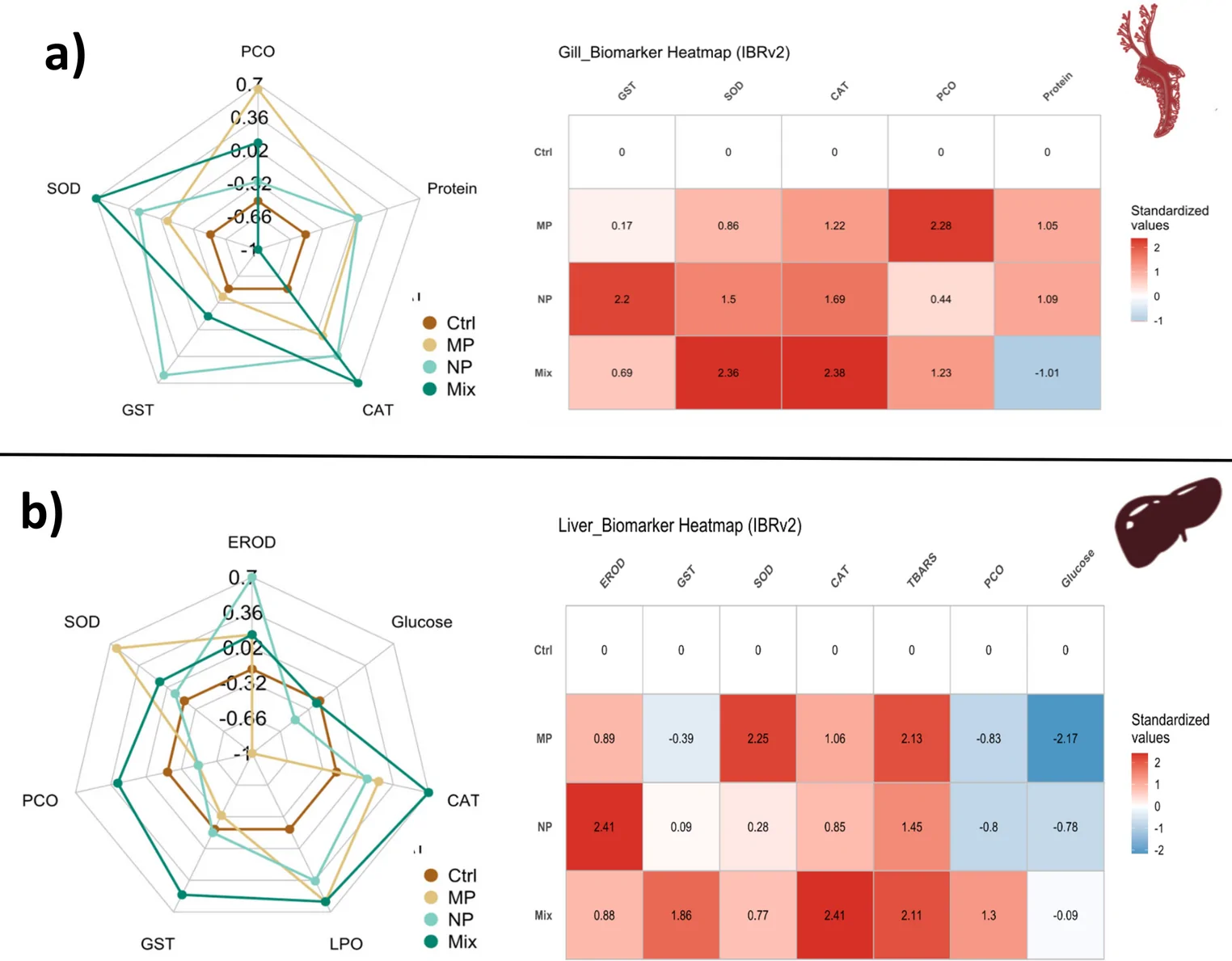
Radar plots of the Integrated Biomarker Response (IBR) indices were generated using the IBRtool package, employing the IBRv2 command based on the methodology proposed by Sanchez et al. (2013)

Figure 4a shows the integrated IBRv2 responses in the gills. The MP group exhibited the highest induction of PCO. The NP group showed pronounced modulation of several biomarkers, including GST, SOD, CAT, and protein levels. The Mix group presented positive responses for SOD, CAT, and PCO, along with negative modulation of total protein content. According to the IBRv2 indices (Table 1), the highest integrated response in the gills was observed in the Mix group, followed by NP and MP.

These results support the hypothesis that combined exposure to PE-MPs and TiO₂ NPs alters the physiological status of tadpole gills and increases the demand on antioxidant defense systems, as evidenced by the PCoA spatial segregation and the positive IBRv2 responses.

As previously discussed, TiO₂ NPs adsorbed onto PE-MPs can retain their ability to generate ROS, although this production may occur locally at the particle surface (Kalčíková et al. 2023). The physicochemical characterization of the particles used in this study, previously reported by Jacintho et al. (2025), supports the occurrence of heteroaggregation and adsorption of TiO₂ NPs onto PE-MP surfaces. This interaction is evidenced by the significant increase in hydrodynamic diameter observed in the mixture (750.3 ± 60.15 nm), compared to isolated PE-MPs (642.5 ± 98.03 nm) and isolated NPs (541.10 ± 50.01 nm), suggesting the formation of larger particle complexes in the aqueous medium.

The shift in zeta potential observed in the mixture (–17.45 ± 2.08 mV) further supports this interpretation (Rozman et al. 2023). The intermediate value, relative to PE-MPs (–13.74 ± 1.06 mV) and TiO₂ NPs (–22.59 ± 1.43 mV), indicates surface modification of microplastics, likely due to partial nanoparticle coating.

In addition, the high polydispersity index (PdI = 0.99 ± 0.07), combined with a zeta potential magnitude below 30 mV, indicates a heterogeneous colloidal system with limited stability. Under these conditions, reduced electrostatic repulsion facilitates particle interactions and aggregation in the aqueous medium (Rozman et al. 2023).

Although such aggregates tend to remain suspended temporarily before precipitating or adhering to aquarium surfaces, part of these complexes may attach to the tadpole body surface, gills, or be ingested. Our results suggest that PE-MP/TiO₂ NP complexes may adhere to and bioaccumulate in the gills, which are highly permeable and vascularized organs with large surface area (Schlenk and De Almeida 2023). This interaction may promote physical contact with epithelial tissues and induce cellular stress through localized ROS production by adsorbed nanoparticles.

Despite this potential interaction, tadpole gills also possess protective mechanisms, such as mucus secretion. In the Mix group, increased SOD and CAT activities were observed without a corresponding increase in PCO, suggesting that antioxidant defenses and mucus-mediated clearance may have contributed to limiting oxidative damage and preserving tissue integrity.

Interestingly, isolated NP exposure also induced antioxidant and detoxification responses. While experimental analyses showed increased SOD activity, the IBRv2 analysis indicated modulation of antioxidant (SOD, CAT) and detoxification (GST) biomarkers.

In addition to direct effects on the gills, these contaminants may also be absorbed and distributed to other organs, such as the liver (Schlenk and De Almeida 2023), which will be discussed in the following section.

Furthermore, aspects of the exposure system may also influence contaminant dynamics. During the experiment, water was partially renewed every 24 h, with 50% replacement using freshly prepared contaminant solutions. However, due to aggregation, precipitation, and bioaccumulation processes, contaminant availability may have varied over time. For example, previously settled aggregates may have been resuspended during water renewal, temporarily increasing contaminant bioavailability. Alternatively, repeated exposure cycles may have progressively increased contaminant body burden before aggregation and sedimentation occurred again.

Therefore, future studies should consider assessing particle kinetics and measuring actual contaminant concentrations in exposure systems to improve understanding of exposure dynamics and toxicokinetic processes.

#### Liver

PERMANOVA also confirmed significant differences among treatments in the liver (*p* = 0.0001). The first two axes explained 56.82% of the total variation (PCoA1 = 30.97%; PCoA2 = 25.85%) (Fig. 3b). Group separation was more pronounced than in the gills, with clearer discrimination among treatments.

Like the gills, the Mix group showed the greatest spatial separation. Vector analysis indicated that this segregation was mainly driven by TBARS, PCO, CAT, and GST. Meanwhile, the MP group showed separation from the Ctrl group, with its positioning positively influenced by the biomarkers SOD, TBARS, and CAT, and a strong negative association with glucose. The MP group was primarily associated with SOD activity, whereas the NP group showed a stronger relationship with EROD and glucose levels.

Figure 4b shows the IBRv2 in the liver. The MP group exhibited increased values of SOD, CAT, and TBARS, along with a more pronounced negative modulation of glucose. The NP group showed an increased response of EROD and TBARS. The Mix group exhibited increased values of GST, CAT, TBARS, and PCO.

According to the IBRv2 indices (Table 1), the highest integrated responses in the liver were observed in the MP and Mix groups, followed by the NP group.

Although the Mix group showed greater spatial separation in the PCoA, indicating a distinct biochemical response profile, the MP group presented slightly higher IBRv2 values (a difference of 0.04 points). This apparent discrepancy occurs because PCoA reflects differences in the pattern of biomarker responses, whereas IBRv2 represents the overall magnitude of stress. In the liver, the MP group exhibited greater metabolic disruption, particularly evidenced by the marked depletion of glucose, which substantially contributed to the integrated stress index. In contrast, the Mix group showed pronounced antioxidant and detoxification responses, but with less severe metabolic impairment, resulting in a slightly lower IBRv2 despite its distinct physiological profile. These differences can also be observed in the biomarker influence pattern in the PCoA, where the MP group is negatively influenced by glucose and positively influenced by SOD, TBARS, and CAT.

In general, our results indicate that the gills, as one of the first points of contact between the organism and contaminants present in the water, trigger faster physiological responses, mainly associated with antioxidant defenses. This pattern is probably related to continuous ventilation movements and mucus restriction, which may facilitate the constant removal of some contaminants from the gill surface, limiting their accumulation and potentially leading to less metabolic disruption. In contrast, the liver, primarily responsible for detoxification and biotransformation, showed clearer metabolic and antioxidant responses. Due to its physiological role and internal location, the liver tends to accumulate contaminants more passively, through systemic circulation (Schlenk and De Almeida 2023). This accumulation can result in more prolonged biochemical responses and lead to moderate to high hepatotoxic effects.

From this perspective, the relationship between our experimental findings and the results obtained from the PCoA and IBRv2 analyses suggests that, during the 15-day exposure period, the organisms intercepted part of the contaminants through the gills. These, in turn, responded via activation of the antioxidant system. Subsequently, part of these pollutants may have been transported into the bloodstream through epithelial crossing via paracellular and transcellular routes (Schlenk and De Almeida 2023), eventually reaching the liver and resulting in detoxification processes and oxidative stress.

In addition, other pathways for pollutant translocation into the bloodstream should be considered, such as the mouth–intestine route, which appears to be one of the most significant pathways for systemic uptake (Araújo et al. 2020). As previously reported by Araújo et al. (2020), PE-MPs of approximately 30 μm have been found bioaccumulated in the liver of *P. cuvieri* tadpoles, and particles up to 48.57 ± 0.929 μm have been detected in the gills.

Finally, based on the experimental results obtained in the liver, we hypothesize that exposure to the pollutants, both individually and especially in the mixture, may have interfered with the Nrf2 expression pathway, one of the key regulators of antioxidant and anti-inflammatory defenses and cellular redox homeostasis, through the activation of genes related to defense enzymes (e.g., SOD, CAT, GST, GPx, and thioredoxin reductase) (Subaramaniyam et al. 2023; Jia et al. 2024). In this context, the increase in TBARS levels observed in all groups, combined with the lack of consistent changes in antioxidant enzyme activities across all groups, may indicate that the interaction of these contaminants interfered with the regulation of this factor, resulting in impairment of cellular defense systems and, consequently, increased lipid peroxidation in all groups.

Similar results were reported by Jia et al. (2024). In that study, the authors found that single and combined exposures to PS NPs (0.025 mg/mL) and TiO₂ NPs (10 mg/kg body weight), in mice, after 28 days of exposure, led to decreased CAT, SOD, GSH, and Nrf2 activity in all exposed groups, along with increased malondialdehyde levels in the mixture-exposed group. On the other hand, LPO products, such as 4-hydroxynonenal, may exert negative feedback on Nrf2, resulting in decreased activity of defense enzymes such as GST.

However, since our results demonstrated an increase in GST activity in the Mix group, it is likely that other, more complex regulatory mechanisms are governing the observed responses. Therefore, we conclude that further targeted studies involving the regulatory pathways of cellular defense systems are needed to better understand the real impacts of these contaminants on the kinetics and regulation of the different factors involved.

Figure 3. Principal Coordinates Analysis (PCoA) based on the Euclidean dissimilarity matrix of biochemical responses in *Aquarana catesbeiana* tadpoles exposed to different treatments: control (Ctrl), polyethylene microplastics (60 mg/L, MP), titanium dioxide nanoparticles (10 µg/L, NP), and the mixture (Mix). Colored regions represent each experimental group. The vectors correspond to the envfit adjustment of response variables, indicating the direction and magnitude of each biomarker’s contribution to the separation among groups. (a) PCoA of tadpole gills; (b) PCoA of tadpole liver.

**Table 1:** Values of the Integrated Biomarker Response indices (IBRv2) corresponding to the control, polyethylene microplastics (60 mg/L, MP), titanium dioxide nanoparticles (10 µg/L, NP), and the combined microplastics with nanoparticles treatments. (Mix)

Group	IBRv2 (Liver)	IBRv2 (Gill)
**Ctrl**	0.000	0.000
**MP**	9.737	5.592
**NP**	6.757	6.923
**Mix**	9.697	7.665
Values were calculated with the “*IBRtools” package*, as proposed by Sanchez et al. (2013)		

## Conclusions

Our experiments showed that the chronic exposure of bullfrog tadpoles to 60 mg/L of PE MPs, either alone or in combination with TiO₂ NPs (10 µg/L), elicited distinct biochemical responses in the gill and liver tissues. In the gills, the combined exposure to MPs and NPs led to increased SOD and CAT activities, suggesting activation of the antioxidant system in response to ROS.

In the liver, increased GST activity was observed in the Mix group and elevated TBARS occurred in all groups. Additionally, glucose levels decreased in the MP group compared to the control, which may indicate disruption of glucose regulatory pathways and could reflect bioaccumulation processes in the tissue.

The IBRv2 analysis for each organ revealed differential biomarker responses depending on the treatment and organ. The indices indicated that the liver showed the highest response values in the MP and Mix groups, suggesting greater susceptibility to stress effects in this organ. In contrast, the gills exhibited smaller deviations among exposures, which may reflect the organ’s ability to translocate xenobiotics into the organism, facilitating greater circulation and dispersal of contaminants.

Overall, this study highlights the importance of evaluating the effects of MPs and NPs both individually and in combination, as well as considering organ-specific responses to chemical stress. Furthermore, it underscores the need for complementary histological and molecular investigations to better understand the mechanisms of bioaccumulation, transport, and toxicity of these contaminants in tadpoles and, by extension, in other organisms.

## Supplementary Information

Below is the link to the electronic supplementary material.

Supplementary Material 1

## Data Availability

The datasets generated and analyzed during the current study are available from the corresponding author on reasonable request.
