# Supplementary material for: Oxidative stress response biomarkers in gills and liver of bullfrog tadpoles semi-chronically exposed to combined microplastics and titanium dioxide nanoparticles
Source: Ecotoxicology. 2026 Jul 2;35(6):128. doi: 10.1007/s10646-026-03110-y (PMC13328142; doi:10.1007/s10646-026-03110-y)
Supplement: Supplementary file 1 — Supplementary Material 1 [file 10646_2026_3110_MOESM1_ESM.docx]

***GILL***

*- SOD (units × mg protein⁻¹) (1 unit of SOD corresponds to the activity capable of inhibiting cytochrome reduction by 50%)*

| Ctrl | MP | NP | Mix |
| --- | --- | --- | --- |
| 38.679 | 46.108 | 46.415 | 47.027 |
| 38.487 | 31.825 | 44.802 | 55.256 |
| 38.679 | 39.335 | 49.589 | 57.581 |
| 32.233 | 31.538 | 40.881 | 48.063 |
| 34.699 | 47.822 | 41.791 | 46.648 |
| 31.704 | 48.118 | 46.577 | 53.448 |

**Mean ± Std. Deviation**

**Ctrl** = 35.7 ± 3.30

**MP** = 40.8 ± 7.74

**NP** = 45.0 ± 3.25

**Mix** = 51.3 ± 4.69

**Dixon test for outliers**

**Ctrl** – Q = 0.075842, p-value = 0.3848

**MP** – Q = 0.01731, p-value = 0.09655

**NP** – Q = 0.34589, p-value = 0.5126

**Mix** – Q = 0.21266, p-value = 0.989

**Shapiro-Wilk normality test**

*W = 0.95546, p-value = 0.3542*

**Levene's Test for Homogeneity of Variance (center = mean)**

*Df F value Pr(>F)*

*group 3 5.6469 0.0057*

*20*

**Modified robust Brown-Forsythe Levene-type test based on the absolute deviations from the median**

Test Statistic = 3.9377, p-value = 0.02332

**Fligner-Killeen test of homogeneity of variances**

Fligner-Killeen:med chi-squared = 7.8188, df = 3, p-value = 0.04991

**studentized Breusch-Pagan test**

BP = 12.569, df = 3, p-value = 0.005668

**Goldfeld-Quandt test**

GQ = 0.46025, df1 = 8, df2 = 8, p-value = 0.8534

group value residuals cooks leverage

1 Ctrl 38.679 2.9321667 1.995785e-02 0.1666667

2 Ctrl 38.487 2.7401667 1.742972e-02 0.1666667

3 Ctrl 38.679 2.9321667 1.995785e-02 0.1666667

4 Ctrl 32.233 -3.5138333 2.866149e-02 0.1666667

5 Ctrl 34.699 -1.0478333 2.548712e-03 0.1666667

6 Ctrl 31.704 -4.0428333 3.794094e-02 0.1666667

7 MP 46.108 5.3170000 6.562506e-02 0.1666667

8 MP 31.825 -8.9660000 1.866095e-01 0.1666667

9 MP 39.335 -1.4560000 4.921066e-03 0.1666667

10 MP 31.538 -9.2530000 1.987474e-01 0.1666667

11 MP 47.822 7.0310000 1.147547e-01 0.1666667

12 MP 48.118 7.3270000 1.246203e-01 0.1666667

13 NP 46.415 1.4058333 4.587796e-03 0.1666667

14 NP 44.802 -0.2071667 9.962679e-05 0.1666667

15 NP 49.589 4.5798333 4.868955e-02 0.1666667

16 NP 40.881 -4.1281667 3.955950e-02 0.1666667

17 NP 41.791 -3.2181667 2.404105e-02 0.1666667

18 NP 46.577 1.5678333 5.706058e-03 0.1666667

19 Mix 47.027 -4.3101667 4.312454e-02 0.1666667

20 Mix 55.256 3.9188333 3.564922e-02 0.1666667

21 Mix 57.581 6.2438333 9.049800e-02 0.1666667

22 Mix 48.063 -3.2741667 2.488502e-02 0.1666667

23 Mix 46.648 -4.6891667 5.104201e-02 0.1666667

24 Mix 53.448 2.1108333 1.034295e-02 0.1666667

**One-way analysis of means (not assuming equal variances)**

F = 14.907, num df = 3.000, denom df = 10.779, p-value = 0.0003732

**Games_howell_test**

# A tibble: 6 × 8

.y. group1 group2 estimate conf.low conf.high p.adj p.adj.signif

* *<chr>* *<chr>* *<chr>* *<dbl>* *<dbl>* *<dbl>* *<dbl>* *<chr>*

1 valor Ctrl MP 5.04 -6.43 16.5 0.503 ns

2 valor Ctrl NP 9.26 3.48 15.0 0.003 **

3 valor Ctrl Mix 15.6 8.27 22.9 0.000444 ***

4 valor MP NP 4.22 -7.26 15.7 0.63 ns

5 valor MP Mix 10.5 -1.21 22.3 0.08 ns

6 valor NP Mix 6.33 -0.964 13.6 0.093 ns

*- PCO (nanomoles of carbonyls × mg protein⁻¹)*

| Ctrl | MP | NP | Mix |
| --- | --- | --- | --- |
| 28.27 | 28.54 | 21.94 | 39.3 |
| 8.9 | 26.72 | 32.65 | 29.39 |
| 5.88 | 50.84 | 32.65 | 31.7 |
| 40.82 | 39.37 | 19.28 | 38.65 |
| 39.18 | 57 | 30.26 | 30.52 |
| 40.15 | 30.94 | 38.14 | 28.5 |

**Mean ± Std. Deviation**

**Ctrl** *= 27.2 ± 16.0*

**MP** *= 38.9 ± 12.6*

**NP** *= 29.2 ± 7.16*

**Mix** *= 33 ± 4.75*

**Dixon test for outliers**

**Ctrl -** Q = 0.086434, p-value = 0.4357

**MP** - Q = 0.20343, p-value = 0.9725

**NP**- Q = 0.14104, p-value = 0.6943

**Mix** - Q = 0.060185, p-value = 0.309

**Shapiro-Wilk normality test**

W = 0.98076, p-value = 0.9094

**Levene's Test for Homogeneity of Variance (center = mean)**

Df F value Pr(>F)

group 3 4.3378 0.01649

20

**Modified robust Brown-Forsythe Levene-type test based on the absolute deviations**

**from the median**

Test Statistic = 2.2508, p-value = 0.1137

**Fligner-Killeen test of homogeneity of variances**

Fligner-Killeen:med chi-squared = 4.6649, df = 3, p-value = 0.198

**studentized Breusch-Pagan test**

BP = 10.156, df = 3, p-value = 0.01729

**Goldfeld-Quandt test**

GQ = 0.17753, df1 = 8, df2 = 8, p-value = 0.9877

group value residuals cooks leverage

1 Ctrl 28.27 1.0700000 0.0005618153 0.1666667

2 Ctrl 8.90 -18.3000000 0.1643342743 0.1666667

3 Ctrl 5.88 -21.3200000 0.2230490473 0.1666667

4 Ctrl 40.82 13.6200000 0.0910290870 0.1666667

5 Ctrl 39.18 11.9800000 0.0704270679 0.1666667

6 Ctrl 40.15 12.9500000 0.0822934953 0.1666667

7 MP 28.54 -10.3616667 0.0526847842 0.1666667

8 MP 26.72 -12.1816667 0.0728181087 0.1666667

9 MP 50.84 11.9383333 0.0699380265 0.1666667

10 MP 39.37 0.4683333 0.0001076307 0.1666667

11 MP 57.00 18.0983333 0.1607322919 0.1666667

12 MP 30.94 -7.9616667 0.0311052684 0.1666667

13 NP 21.94 -7.2133333 0.0255327725 0.1666667

14 NP 32.65 3.4966667 0.0059997677 0.1666667

15 NP 32.65 3.4966667 0.0059997677 0.1666667

16 NP 19.28 -9.8733333 0.0478358583 0.1666667

17 NP 30.26 1.1066667 0.0006009795 0.1666667

18 NP 38.14 8.9866667 0.0396299239 0.1666667

19 Mix 39.30 6.2900000 0.0194145471 0.1666667

20 Mix 29.39 -3.6200000 0.0064304759 0.1666667

21 Mix 31.70 -1.3100000 0.0008421095 0.1666667

22 Mix 38.65 5.6400000 0.0156093270 0.1666667

23 Mix 30.52 -2.4900000 0.0030424585 0.1666667

24 Mix 28.50 -4.5100000 0.0099811149 0.1666667

**One-way analysis of means (not assuming equal variances)**

F = 1.0477, num df = 3.000, denom df = 10.255, p-value = 0.4125

*- GST (µmoles of thioether formed × min⁻¹ × mg protein⁻¹)*

| Ctrl | MP | NP | Mix |
| --- | --- | --- | --- |
| 0.052 | 0.084 | 0.054 | 0.063 |
| 0.063 | 0.048 | 0.054 | 0.072 |
| 0.083 | 0.053 | 0.065 | 0.064 |
| 0.08 | 0.059 | 0.072 | 0.134 |
| 0.074 | 0.085 | 0.076 | 0.071 |
| 0.028 | 0.052 | 0.072 | 0.064 |
| 0.052 | 0.084 | 0.054 | 0.063 |

**Mean ± Std. Deviation**

**Ctrl** = 0.063 ± 0.021

**MP** = 0.064 ± 0.017

**NP** = 0.066 ± 0.01

**Mix** = 0.064 ± 0.008

**Dixon test for outliers**

**Ctrl -** Q = 0.43636, p-value = 0.2838

**MP** - Q = 0.027027, p-value = 0.1454

**NP**- Q = 0.5, p-value = 0.1725

**Mix** - Q = 0.59091, p-value = 0.07294

**Shapiro-Wilk normality test**

W = 0.95872, p-value = 0.4133

**Levene's Test for Homogeneity of Variance (center = mean)**

Df F value Pr(>F)

group 3 3.0912 0.05034 .

20

**Modified robust Brown-Forsythe Levene-type test based on the absolute deviations**

**from the median**

Test Statistic = 1.7486, p-value = 0.1894

**Fligner-Killeen test of homogeneity of variances**

Fligner-Killeen:med chi-squared = 5.0719, df = 3, p-value = 0.1666

**studentized Breusch-Pagan test**

BP = 5.8253, df = 3, p-value = 0.1204

**Goldfeld-Quandt test**

GQ = 0.17665, df1 = 8, df2 = 8, p-value = 0.9879

group valor residuals cooks leverage

1 Ctrl 0.052 -1.133333e-02 3.699496e-02 0.1666667

2 Ctrl 0.063 -3.333333e-04 3.200256e-05 0.1666667

3 Ctrl 0.083 1.966667e-02 1.114009e-01 0.1666667

4 Ctrl 0.080 1.666667e-02 8.000640e-02 0.1666667

5 Ctrl 0.074 1.066667e-02 3.277062e-02 0.1666667

6 Ctrl 0.028 -3.533333e-02 3.595808e-01 0.1666667

7 MP 0.084 2.050000e-02 1.210417e-01 0.1666667

8 MP 0.048 -1.550000e-02 6.919754e-02 0.1666667

9 MP 0.053 -1.050000e-02 3.175454e-02 0.1666667

10 MP 0.059 -4.500000e-03 5.832467e-03 0.1666667

11 MP 0.085 2.150000e-02 1.331387e-01 0.1666667

12 MP 0.052 -1.150000e-02 3.809105e-02 0.1666667

13 NP 0.072 3.500000e-03 3.528282e-03 0.1666667

14 NP 0.054 -1.450000e-02 6.055684e-02 0.1666667

15 NP 0.065 -3.500000e-03 3.528282e-03 0.1666667

16 NP 0.072 3.500000e-03 3.528282e-03 0.1666667

17 NP 0.076 7.500000e-03 1.620130e-02 0.1666667

18 NP 0.072 3.500000e-03 3.528282e-03 0.1666667

19 Mix 0.063 -1.000000e-03 2.880230e-04 0.1666667

20 Mix 0.072 8.000000e-03 1.843347e-02 0.1666667

21 Mix 0.064 1.517883e-18 6.635961e-34 0.1666667

22 Mix 0.050 -1.400000e-02 5.645252e-02 0.1666667

23 Mix 0.071 7.000000e-03 1.411313e-02 0.1666667

24 Mix 0.064 1.517883e-18 6.635961e-34 0.1666667

**One-way analysis of means**

F = 0.027067, num df = 3, denom df = 20, p-value = 0.9938

*- CAT (micromoles of H₂O₂ degraded × min⁻¹ × mg protein⁻¹)*

| Ctrl | MP | NP | Mix |
| --- | --- | --- | --- |
| 0.059 | 0.05 | 0.048 | 0.061 |
| 0.037 | 0.062 | 0.055 | 0.097 |
| 0.068 | 0.053 | 0.072 | 0.065 |
| 0.031 | 0.078 | 0.096 | 0.08 |
| 0.036 | 0.044 | 0.048 | 0.081 |
| 0.059 | 0.08 | 0.082 | 0.074 |

**Mean ± Std. Deviation**

***Ctrl*** *= 0.048 ± 0.015*

***MP*** *= 0.061 ± 0.015*

***NP*** *= 0.067 ± 0.02*

***Mix*** *= 0.076 ± 0.013*

**Dixon test for outliers**

**Ctrl -** Q = 0.24324, p-value = 0.8672

**MP** - Q = 0.055556, p-value = 0.2864

**NP**- Q = 0.29167, p-value = 0.689

**Mix** - Q = 0.44444, p-value = 0.2675

**Shapiro-Wilk normality test**

W = 0.91812, p-value = 0.05308

**Levene's Test for Homogeneity of Variance (center = mean)**

Df F value Pr(>F)

group 3 1.0825 0.3792

20

**Modified robust Brown-Forsythe Levene-type test based on the absolute deviations**

**from the median**

Test Statistic = 0.92803, p-value = 0.4454

**Fligner-Killeen test of homogeneity of variances**

Fligner-Killeen:med chi-squared = 1.7509, df = 3, p-value = 0.6257

**studentized Breusch-Pagan test**

BP = 3.3105, df = 3, p-value = 0.3462

**Goldfeld-Quandt test**

GQ = 1.2017, df1 = 8, df2 = 8, p-value = 0.4006

group value residuals cooks leverage

1 Ctrl 0.059 0.0106666667 0.0267275693 0.1666667

2 Ctrl 0.037 -0.0113333333 0.0301729201 0.1666667

3 Ctrl 0.068 0.0196666667 0.0908580750 0.1666667

4 Ctrl 0.031 -0.0173333333 0.0705774878 0.1666667

5 Ctrl 0.036 -0.0123333333 0.0357324633 0.1666667

6 Ctrl 0.059 0.0106666667 0.0267275693 0.1666667

7 MP 0.050 -0.0111666667 0.0292920065 0.1666667

8 MP 0.062 0.0008333333 0.0001631321 0.1666667

9 MP 0.053 -0.0081666667 0.0156672104 0.1666667

10 MP 0.078 0.0168333333 0.0665644372 0.1666667

11 MP 0.044 -0.0171666667 0.0692267537 0.1666667

12 MP 0.080 0.0188333333 0.0833213703 0.1666667

13 NP 0.048 -0.0188333333 0.0833213703 0.1666667

14 NP 0.055 -0.0118333333 0.0328939641 0.1666667

15 NP 0.072 0.0051666667 0.0062707993 0.1666667

16 NP 0.096 0.0291666667 0.1998368679 0.1666667

17 NP 0.048 -0.0188333333 0.0833213703 0.1666667

18 NP 0.082 0.0151666667 0.0540358891 0.1666667

19 Mix 0.061 -0.0153333333 0.0552300163 0.1666667

20 Mix 0.097 0.0206666667 0.1003327896 0.1666667

21 Mix 0.065 -0.0113333333 0.0301729201 0.1666667

22 Mix 0.080 0.0036666667 0.0031582382 0.1666667

23 Mix 0.081 0.0046666667 0.0051158238 0.1666667

24 Mix 0.074 -0.0023333333 0.0012789560 0.1666667

**One-way analysis of means**

F = 3.217, num df = 3, denom df = 20, p-value = 0.04475

**Tuckey test**

# A tibble: 6 × 9

term group1 group2 null.value estimate conf.low conf.high p.adj p.adj.signif

* *<chr>* *<chr>* *<chr>* *<dbl>* *<dbl>* *<dbl>* *<dbl>* *<dbl>* *<chr>*

1 grupo Ctrl MP 0 0.0128 -0.0130 0.0387 0.519 ns

2 grupo Ctrl NP 0 0.0185 -0.00733 0.0443 0.219 ns

3 grupo Ctrl Mix 0 0.0280 0.00217 0.0538 0.0306 *

4 grupo MP NP 0 0.00567 -0.0202 0.0315 0.926 ns

5 grupo MP Mix 0 0.0152 -0.0107 0.0410 0.378 ns

6 grupo NP Mix 0 0.0095 -0.0163 0.0353 0.734 ns

*- Protein (mg of protein × mg of gill mass⁻¹)*

| Ctrl | MP | NP | Mix |
| --- | --- | --- | --- |
| 0.042 | 0.029 | 0.026 | 0.027 |
| 0.035 | 0.048 | 0.044 | 0.011 |
| 0.017 | 0.025 | 0.047 | 0.027 |
| 0.037 | 0.041 | 0.053 | 0.031 |
| 0.046 | 0.053 | 0.027 | 0.031 |
| 0.013 | 0.044 | 0.045 | 0.025 |

**Mean ± Std. Deviation**

**Ctrl** = 0.032 ± 0.014

**MP** = 0.04 ± 0.011

**NP** = 0.04 ± 0.011

**Mix** = 0.025 ± 0.007

**Dixon test for outliers**

**Ctrl -** Q = 0.12121, p-value = 0.601

**MP** - Q = 0.14286, p-value = 0.7027

**NP**- Q = 0.037037, p-value = 0.1952

**Mix** - Q = 0.7, p-value = 0.01944

alternative hypothesis: lowest value 0.011 is an outlier

**Shapiro-Wilk normality test**

W = 0.88144, p-value = 0.008883

**Levene's Test for Homogeneity of Variance (center = mean)**

Df F value Pr(>F)

group 3 1.4448 0.2596

20

**Modified robust Brown-Forsythe Levene-type test based on the absolute deviations from the median**

Test Statistic = 0.58616, p-value = 0.6311

**Fligner-Killeen test of homogeneity of variances**

Fligner-Killeen:med chi-squared = 2.5941, df = 3, p-value = 0.4585

**studentized Breusch-Pagan test**

BP = 3.6532, df = 3, p-value = 0.3014

**Goldfeld-Quandt test**

GQ = 0.59462, df1 = 8, df2 = 8, p-value = 0.7608

group value residuals cooks leverage value_wins

*<fct>* *<dbl>* *<dbl>* *<dbl>* *<dbl>* *<dbl>*

1 Ctrl 0.042 0.0103 0.0532 0.167 0.042

2 Ctrl 0.035 0.00333 0.00553 0.167 0.035

3 Ctrl 0.017 -0.0147 0.107 0.167 0.017

4 Ctrl 0.037 0.00533 0.0142 0.167 0.037

5 Ctrl 0.046 0.0143 0.102 0.167 0.045

6 Ctrl 0.013 -0.0187 0.173 0.167 0.014

7 MP 0.029 -0.011 0.0602 0.167 0.029

8 MP 0.048 0.008 0.0319 0.167 0.048

9 MP 0.025 -0.015 0.112 0.167 0.026

10 MP 0.041 0.00100 0.000498 0.167 0.041

11 MP 0.053 0.013 0.0841 0.167 0.0518

12 MP 0.044 0.00400 0.00797 0.167 0.044

13 NP 0.026 -0.0143 0.102 0.167 0.0262

14 NP 0.044 0.00367 0.00669 0.167 0.044

15 NP 0.047 0.00667 0.0221 0.167 0.047

16 NP 0.053 0.0127 0.0799 0.167 0.0515

17 NP 0.027 -0.0133 0.0885 0.167 0.027

18 NP 0.045 0.00467 0.0108 0.167 0.045

19 Mix 0.027 0.00167 0.00138 0.167 0.027

20 Mix 0.011 -0.0143 0.102 0.167 0.046

21 Mix 0.027 0.00167 0.00138 0.167 0.027

22 Mix 0.031 0.00567 0.0160 0.167 0.031

23 Mix 0.031 0.00567 0.0160 0.167 0.031

24 Mix 0.025 -0.000333 0.0000553 0.167 0.0255

**kruskal_test (values_wins ~ group)**

# A tibble: 1 × 6

.y. n statistic df p method

* *<chr>* *<int>* *<dbl>* *<int>* *<dbl>* *<chr>*

1 values_wins 24 5.64 3 0.131 Kruskal-Wallis

**kruskal_effsize(values_wins ~ group)**

# A tibble: 1 × 5

.y. n effsize method magnitude

* *<chr>* *<int>* *<dbl>* *<chr>* *<ord>*

1 values_wins 24 0.132 eta2[H] moderate

**Permanova e PCoA:**

Permutation test for adonis under reduced model

Terms added sequentially (first to last)

Permutation: free

Number of permutations: 10000

adonis2(formula = vars.scaled ~ Group, data = dados, permutations = 10000, method = "euclidean")

Df SumOfSqs R2 F Pr(>F)

Group 3 31.653 0.27524 2.5318 0.0014 **

Residual 20 83.347 0.72476

Total 23 115.000 1.00000

---

Signif. codes: 0 ‘***’ 0.001 ‘**’ 0.01 ‘*’ 0.05 ‘.’ 0.1 ‘ ’ 1

*****VECTORS**

Dim1 Dim2 r2 Pr(>r)

PCO 0.86522 -0.50140 0.1967 0.1049895

SOD -0.87595 -0.48241 0.7292 9.999e-05 ***

GST -0.09901 -0.99509 0.7182 9.999e-05 ***

CAT -0.99673 0.08080 0.5505 0.0005000 ***

Protein 0.61731 -0.78672 0.4979 0.0007999 ***

---

Signif. codes: 0 ‘***’ 0.001 ‘**’ 0.01 ‘*’ 0.05 ‘.’ 0.1 ‘ ’ 1

Permutation: free

Number of permutations: 10000

***LIVER***

*- EROD (pmol resorufin min^-1^ mg protein^-1^)*

| Ctrl | MP | NP | Mix |
| --- | --- | --- | --- |
| 6.79 | 13.97 | 19.38 | 8.1 |
| 8.51 | 6.54 | 11.23 | 9.7 |
| 9.94 | 19.22 | 8.02 | 10.62 |
| 9.84 | 9.82 | 27.31 | 8.06 |
| 9.8 | 6.44 | 15.44 | 17.23 |
| 12.64 | 12.86 | 12.2 | 14.97 |

**Mean ± Std. Deviation**

group variable n mean sd

*<fct>* *<fct>* *<dbl>* *<dbl>* *<dbl>*

1 Ctrl value 6 9.59 1.93

2 MP value 6 11.5 4.91

3 NP value 6 15.6 6.92

4 Mix value 6 11.4 3.80

**Dixon test for outliers**

**Ctrl -** Q = 0.46154, p-value = 0.2351

**MP** - Q = 0.4108, p-value = 0.3395

**NP**- Q = 0.41109, p-value = 0.3388

**Mix** - Q = 0.24646, p-value = 0.8549

| **Shapiro-Wilk normality test**  W = 0.95872, p-value = 0.4132  **Levene's Test for Homogeneity of Variance (center = mean)**  Df F value Pr(>F)  group 3 2.3677 0.1012  20  **Modified robust Brown-Forsythe Levene-type test based on the**  **absolute deviations from the median**  Test Statistic = 1.851, p-value = 0.1705   \|  \| \| --- \| |
| --- | --- |

**Fligner-Killeen test of homogeneity of variances**

Fligner-Killeen:med chi-squared = 5.1218, df = 3, p-value = 0.1631

**studentized Breusch-Pagan test**

BP = 5.096, df = 3, p-value = 0.1649

**Goldfeld-Quandt test**

GQ = 2.2415, df1 = 8, df2 = 8, p-value = 0.1373

grupo valor residuos cooks leverage

1 Ctrl 6.79 -2.7966667 2.081405e-02 0.1666667

2 Ctrl 8.51 -1.0766667 3.084876e-03 0.1666667

3 Ctrl 9.94 0.3533333 3.322343e-04 0.1666667

4 Ctrl 9.84 0.2533333 1.707890e-04 0.1666667

5 Ctrl 9.80 0.2133333 1.211135e-04 0.1666667

6 Ctrl 12.64 3.0533333 2.480982e-02 0.1666667

7 MP 13.97 2.4950000 1.656595e-02 0.1666667

8 MP 6.54 -4.9350000 6.481113e-02 0.1666667

9 MP 19.22 7.7450000 1.596313e-01 0.1666667

10 MP 9.82 -1.6550000 7.289055e-03 0.1666667

11 MP 6.44 -5.0350000 6.746433e-02 0.1666667

12 MP 12.86 1.3850000 5.104754e-03 0.1666667

13 NP 19.38 3.7833333 3.809118e-02 0.1666667

14 NP 11.23 -4.3666667 5.074291e-02 0.1666667

15 NP 8.02 -7.5766667 1.527677e-01 0.1666667

16 NP 27.31 11.7133333 3.651205e-01 0.1666667

17 NP 15.44 -0.1566667 6.531733e-05 0.1666667

18 NP 12.20 -3.3966667 3.070302e-02 0.1666667

19 Mix 8.10 -3.3466667 2.980576e-02 0.1666667

20 Mix 9.70 -1.7466667 8.118865e-03 0.1666667

21 Mix 10.62 -0.8266667 1.818595e-03 0.1666667

22 Mix 8.06 -3.3866667 3.052250e-02 0.1666667

23 Mix 17.23 5.7833333 8.900854e-02 0.1666667

24 Mix 14.97 3.5233333 3.303564e-02 0.1666667

**ANOVA**

Df Sum Sq Mean Sq F value Pr(>F)

group 3 116.0 38.68 1.716 0.196

Residuals 20 450.9 22.55

*- SOD (units × mg protein⁻¹) (1 unit of SOD corresponds to the activity capable of inhibiting cytochrome reduction by 50%)*

| Ctrl | MP | NP | Mix |
| --- | --- | --- | --- |
| 31.27 | 32.1 | 43.45 | 31.94 |
| 43.43 | 89.6 | 35.84 | 65.42 |
| 37.27 | 87.5 | 52.16 | 53.65 |
| 39.72 | 61.38 | 28.36 | 55.34 |
| 29.2 | 64.55 | 55.37 | 41.18 |
| 50.22 | 37.23 | 35.16 | 39.9 |

**Mean ± Std. Deviation**

**Ctrl** = 38.5 ± 7.79

**MP** = 62.1± 24.2

**NP** = 41.7 ± 10.5

**Mi**x = 47.9 ± 12.3

**Dixon test for outliers**

**Ctrl -** Q = 0.32303, p-value = 0.5837

**MP** - Q = 0.089217, p-value = 0.4491

**NP**- Q = 0.11884, p-value = 0.5899

**Mix** - Q = 0.30108, p-value = 0.6565

**Shapiro-Wilk normality test**

W = 0.98483, p-value = 0.9658

**Levene's Test for Homogeneity of Variance (center = mean)**

Df F value Pr(>F)

group 3 2.9102 0.05974 .

20

**Modified robust Brown-Forsythe Levene-type test based on the**

**absolute deviations from the median**

Test Statistic = 2.8345, p-value = 0.06422

**Fligner-Killeen test of homogeneity of variances**

Fligner-Killeen:med chi-squared = 5.5188, df = 3, p-value = 0.1375

**studentized Breusch-Pagan test**

BP = 10.946, df = 3, p-value = 0.01202

**Goldfeld-Quandt test**

GQ = 0.40672, df1 = 8, df2 = 8, p-value = 0.8876

group value residuals cooks leverage

1 Ctrl 31.27 -7.248333 0.0138760369 0.1666667

2 Ctrl 43.43 4.911667 0.0063715765 0.1666667

3 Ctrl 37.27 -1.248333 0.0004115762 0.1666667

4 Ctrl 39.72 1.201667 0.0003813793 0.1666667

5 Ctrl 29.20 -9.318333 0.0229332504 0.1666667

6 Ctrl 50.22 11.701667 0.0361646757 0.1666667

7 MP 32.10 -29.960000 0.2370678976 0.1666667

8 MP 89.60 27.540000 0.2003166285 0.1666667

9 MP 87.50 25.440000 0.1709319882 0.1666667

10 MP 61.38 -0.680000 0.0001221257 0.1666667

11 MP 64.55 2.490000 0.0016375246 0.1666667

12 MP 37.23 -24.830000 0.1628330543 0.1666667

13 NP 43.45 1.726667 0.0007874195 0.1666667

14 NP 35.84 -5.883333 0.0091418910 0.1666667

15 NP 52.16 10.436667 0.0287682044 0.1666667

16 NP 28.36 -13.363333 0.0471648799 0.1666667

17 NP 55.37 13.646667 0.0491860897 0.1666667

18 NP 35.16 -6.563333 0.0113772697 0.1666667

19 Mix 31.94 -15.965000 0.0673173445 0.1666667

20 Mix 65.42 17.515000 0.0810232041 0.1666667

21 Mix 53.65 5.745000 0.0087170432 0.1666667

22 Mix 55.34 7.435000 0.0145999402 0.1666667

23 Mix 41.18 -6.725000 0.0119446577 0.1666667

24 Mix 39.90 -8.005000 0.0169243424 0.1666667

**One-way analysis of means (not assuming equal variances)**

F = 1.977, num df = 3.00, denom df = 10.64, p-value = 0.1778

*- PCO (nanomoles of carbonyls × mg protein⁻¹)*

| Ctrl | MP | NP | Mix |
| --- | --- | --- | --- |
| 39.059 | 21.5 | 18.426 | 62.809 |
| 35.851 | 27.43 | 30.516 | 74.912 |
| 26.072 | 21.696 | 31.578 | 41.422 |
| 22.734 | 20.312 | 37.138 | 36.524 |
| 42.614 | 30.762 | 11.292 | 42.43 |
| 38.851 | 18.085 | 12.578 | 117.114 |

**Mean ± Std. Deviation**

**Ctrl** = 34.2 ± 7.95

**MP** = 23.3 ± 4.79

**NP** = 23.6 ± 10.9

**Mi**x = 62.5±30.5

**Dixon test for outliers**

**Ctrl -** Q = 0.16791, p-value = 0.8176

**MP** - Q = 0.26284, p-value = 0.7932

**NP**- Q = 0.21512, p-value = 0.9789

**Mix** - Q = 0.52366, p-value = 0.1405

**Shapiro-Wilk normality test**

W = 0.86417, p-value = 0.004053

**Levene's Test for Homogeneity of Variance (center = mean)**

Df F value Pr(>F)

group 3 4.5401 0.0139 *

20

**Modified robust Brown-Forsythe Levene-type test based on the absolute deviations**

**from the median**

Test Statistic = 3.4038, p-value = 0.03766

**Fligner-Killeen test of homogeneity of variances**

Fligner-Killeen:med chi-squared = 11.124, df = 3, p-value = 0.01107

**studentized Breusch-Pagan test**

BP = 6.6467, df = 3, p-value = 0.08405

**Goldfeld-Quandt test**

GQ = 12.189, df1 = 8, df2 = 8, p-value = 0.0009591

alternative hypothesis: variance increases from segment 1 to 2

grupo valor residuos cooks leverage

1 Ctrl 39.059 4.8623333 4.991216e-03 0.1666667

2 Ctrl 35.851 1.6543333 5.777806e-04 0.1666667

3 Ctrl 26.072 -8.1246667 1.393567e-02 0.1666667

4 Ctrl 22.734 -11.4626667 2.773883e-02 0.1666667

5 Ctrl 42.614 8.4173333 1.495774e-02 0.1666667

6 Ctrl 38.850 4.6533333 4.571358e-03 0.1666667

7 MP 21.500 -1.7975000 6.821104e-04 0.1666667

8 MP 27.430 4.1325000 3.605310e-03 0.1666667

9 MP 21.696 -1.6015000 5.414655e-04 0.1666667

10 MP 20.312 -2.9855000 1.881703e-03 0.1666667

11 MP 30.762 7.4645000 1.176301e-02 0.1666667

12 MP 18.085 -5.2125000 5.735998e-03 0.1666667

13 NP 18.426 -5.1620000 5.625393e-03 0.1666667

14 NP 30.516 6.9280000 1.013287e-02 0.1666667

15 NP 31.578 7.9900000 1.347753e-02 0.1666667

16 NP 37.138 13.5500000 3.876105e-02 0.1666667

17 NP 11.292 -12.2960000 3.191866e-02 0.1666667

18 NP 12.578 -11.0100000 2.559125e-02 0.1666667

19 Mix 62.809 0.2738333 1.583031e-05 0.1666667

20 Mix 74.912 12.3768333 3.233970e-02 0.1666667

21 Mix 41.422 -21.1131667 9.410737e-02 0.1666667

22 Mix 36.524 -26.0111667 1.428356e-01 0.1666667

23 Mix 42.430 -20.1051667 8.533599e-02 0.1666667

24 Mix 117.114 54.5788333 6.288765e-01 0.1666667

**λ** = -0.06060606

**Shapiro-Wilk normality test (λ)**

W = 0.98609, p-value = 0.9772

**Levene's Test for Homogeneity of Variance (center = mean) (λ)**

Df F value Pr(>F)

group 3 4.3395 0.01647 *

20

**Modified robust Brown-Forsythe Levene-type test based on the absolute deviations**

**from the median (λ)**

Test Statistic = 3.0265, p-value = 0.0535

**Fligner-Killeen test of homogeneity of variances (λ)**

Fligner-Killeen:med chi-squared = 6.6046, df = 3, p-value = 0.08563

**studentized Breusch-Pagan test (λ)**

BP = 8.6105, df = 3, p-value = 0.03494

**Goldfeld-Quandt test (λ)**

GQ = 4.3292, df1 = 8, df2 = 8, p-value = 0.02674

alternative hypothesis: variance increases from segment 1 to 2

grupo valor residuos cooks leverage valor_λ

1 Ctrl 39.059 4.8623333 0.010777464 0.1666667 3.286557

2 Ctrl 35.851 1.6543333 0.002331230 0.1666667 3.217747

3 Ctrl 26.072 -8.1246667 0.025807292 0.1666667 2.958861

4 Ctrl 22.734 -11.4626667 0.063383624 0.1666667 2.845960

5 Ctrl 42.614 8.4173333 0.025588358 0.1666667 3.356132

6 Ctrl 38.850 4.6533333 0.010069427 0.1666667 3.282260

7 MP 21.500 -1.7975000 0.001748550 0.1666667 2.799699

8 MP 27.430 4.1325000 0.014450504 0.1666667 3.000467

9 MP 21.696 -1.6015000 0.001277079 0.1666667 2.807232

10 MP 20.312 -2.9855000 0.006395253 0.1666667 2.752422

11 MP 30.762 7.4645000 0.038276499 0.1666667 3.093937

12 MP 18.085 -5.2125000 0.025068966 0.1666667 2.655323

13 NP 18.426 -5.1620000 0.008750267 0.1666667 2.670988

14 NP 30.516 6.9280000 0.058818271 0.1666667 3.087412

15 NP 31.578 7.9900000 0.070195212 0.1666667 3.115192

16 NP 37.138 13.5500000 0.137342305 0.1666667 3.246108

17 NP 11.292 -12.2960000 0.184653812 0.1666667 2.254436

18 NP 12.578 -11.0100000 0.125889639 0.1666667 2.347251

19 Mix 62.809 0.2738333 0.003722154 0.1666667 3.661540

20 Mix 74.912 12.3768333 0.029266299 0.1666667 3.797922

21 Mix 41.422 -21.1131667 0.041501701 0.1666667 3.333512

22 Mix 36.524 -26.0111667 0.081265124 0.1666667 3.232710

23 Mix 42.430 -20.1051667 0.035437024 0.1666667 3.352684

24 Mix 117.114 54.5788333 0.197983946 0.1666667 4.137289

**One-way analysis of means (not assuming equal variances) (λ)**

F = 7.972, num df = 3.000, denom df = 10.566, p-value = 0.004616

**Games_howell_test (λ)**

# A tibble: 6 × 8

.y. group1 group2 estimate conf.low conf.high p.adj p.adj.signif

* *<chr>* *<chr>* *<chr>* *<dbl>* *<dbl>* *<dbl>* *<dbl>* *<chr>*

1 valor_bc Ctrl MP -0.306 -0.638 0.0251 0.072 ns

2 valor_bc Ctrl NP -0.371 -1.00 0.261 0.296 ns

3 valor_bc Ctrl Mix 0.428 -0.0958 0.952 0.115 ns

4 valor_bc MP NP -0.0646 -0.693 0.564 0.984 ns

5 valor_bc MP Mix 0.734 0.220 1.25 0.009 **

6 valor_bc NP Mix 0.799 0.111 1.49 0.023 *

*- GST (µmoles of thioether formed × min⁻¹ × mg protein⁻¹)*

| Ctrl | MP | NP | Mix |
| --- | --- | --- | --- |
| 0.2735 | 0.2073 | 0.3156 | 0.5027 |
| 0.3097 | 0.2335 | 0.2508 | 0.4304 |
| 0.2362 | 0.125 | 0.2162 | 0.3707 |
| 0.3212 | 0.3137 | 0.2787 | 0.5634 |
| 0.1499 | 0.1614 | 0.2502 | 0.3355 |
| 0.1949 | 0.2644 | 0.2186 | 0.5565 |

**Mean ± Std. Deviation**

**Ctrl** = 0.248 ± 0.067

**MP** = 0.218 ± 0.069

**NP** = 0.255 ± 0.038

**Mi**x = 0.46 ± 0.096

**Dixon test for outliers**

**Ctrl -** Q = 0.2627, p-value = 0.7937

**MP** - Q = 0.26126, p-value = 0.799

**NP**- Q = 0.37123, p-value = 0.4396

**Mix** - Q = 0.15445, p-value = 0.7564

**Shapiro-Wilk normality test**

W = 0.96774, p-value = 0.6117

**Levene's Test for Homogeneity of Variance (center = mean)**

Df F value Pr(>F)

group 3 2.6921 0.07366 .

20

**Modified robust Brown-Forsythe Levene-type test based on the**

**absolute deviations from the median**

Test Statistic = 2.678, p-value = 0.07467

**Fligner-Killeen test of homogeneity of variances**

Fligner-Killeen:med chi-squared = 6.811, df = 3, p-value = 0.07817

**studentized Breusch-Pagan test**

BP = 7.173, df = 3, p-value = 0.06658

**Goldfeld-Quandt test**

GQ = 1.1605, df1 = 8, df2 = 8, p-value = 0.4192

group value residuals cooks leverage

1 Ctrl 0.2735 0.025933333 0.0081357615 0.1666667

2 Ctrl 0.3097 0.062133333 0.0467015003 0.1666667

3 Ctrl 0.2362 -0.011366667 0.0015629597 0.1666667

4 Ctrl 0.3212 0.073633333 0.0655889152 0.1666667

5 Ctrl 0.1499 -0.097666667 0.1153916165 0.1666667

6 Ctrl 0.1949 -0.052666667 0.0335546869 0.1666667

7 MP 0.2073 -0.010250000 0.0012709523 0.1666667

8 MP 0.2335 0.015950000 0.0030775343 0.1666667

9 MP 0.1250 -0.092550000 0.1036178012 0.1666667

10 MP 0.3137 0.096150000 0.1118356080 0.1666667

11 MP 0.1614 -0.056150000 0.0381400315 0.1666667

12 MP 0.2644 0.046850000 0.0265522126 0.1666667

13 NP 0.3156 0.060583333 0.0444004995 0.1666667

14 NP 0.2508 -0.004216667 0.0002150899 0.1666667

15 NP 0.2162 -0.038816667 0.0182271179 0.1666667

16 NP 0.2787 0.023683333 0.0067852707 0.1666667

17 NP 0.2502 -0.004816667 0.0002806562 0.1666667

18 NP 0.2186 -0.036416667 0.0160428642 0.1666667

19 Mix 0.5027 0.042833333 0.0221944952 0.1666667

20 Mix 0.4304 -0.029466667 0.0105037299 0.1666667

21 Mix 0.3707 -0.089166667 0.0961804023 0.1666667

22 Mix 0.5634 0.103533333 0.1296707191 0.1666667

23 Mix 0.3355 -0.124366667 0.1871067756 0.1666667

24 Mix 0.5565 0.096633333 0.1129627996 0.1666667

**One-way analysis of means**

F = 14.931, num df = 3, denom df = 20, p-value = 2.464e-05

**Tuckey test**

| # A tibble: 6 × 9  term group1 group2 null.value estimate conf.low conf.high p.adj p.adj.signif  * *<chr>* *<chr>* *<chr>* *<dbl>* *<dbl>* *<dbl>* *<dbl>* *<dbl>* *<chr>*  1 grupo Ctrl MP 0 -0.0300 -0.144 0.0838 0.881 ns  2 grupo Ctrl NP 0 0.00745 -0.106 0.121 0.998 ns  3 grupo Ctrl Mix 0 0.212 0.0985 0.326 0.000225 ***  4 grupo MP NP 0 0.0375 -0.0763 0.151 0.794 ns  5 grupo MP Mix 0 0.242 0.129 0.356 0.0000438 ****  6 grupo NP Mix 0 0.205 0.0910 0.319 0.00034 *** |
| --- |
| \| *- LPO (μM TBARS mg protein^-1^)*   \| Ctrl \| MP \| NP \| Mix \| \| --- \| --- \| --- \| --- \| \| 8.056 \| 38.99 \| 25.575 \| 30.565 \| \| 9.219 \| 44.7 \| 32.044 \| 21.682 \| \| 15.762 \| 39.826 \| 22.377 \| 29.544 \| \| 8.48 \| 36.079 \| 22.407 \| 42.18 \| \| 12.004 \| 17.497 \| 17.916 \| 28.506 \| \| 15.461 \| 12.325 \| 17.002 \| 35.354 \|   **Mean ± Std. Deviation**  **Ctrl** = 11.5 ± 3.47  **MP** = 31.6 ± 13.3  **NP** = 22.9 ± 5.49  **Mi**x = 31.3 ± 6.91  **Dixon test for outliers**  **Ctrl -** Q = 0.03906, p-value = 0.2053  **MP** - Q = 0.15975, p-value = 0.7807  **NP**- Q = 0.43006, p-value = 0.2969  **Mix** - Q = 0.33301, p-value = 0.5521  **Shapiro-Wilk normality test**  W = 0.96607, p-value = 0.5717  **Levene's Test for Homogeneity of Variance (center = mean)**  Df F value Pr(>F)  group 3 5.386 0.006987 **  20  **Modified robust Brown-Forsythe Levene-type test based on the**  **absolute deviations from the median**  Test Statistic = 1.4516, p-value = 0.2577  **Fligner-Killeen test of homogeneity of variances**  Fligner-Killeen:med chi-squared = 2.4955, df = 3, p-value = 0.4761  **studentized Breusch-Pagan test**  BP = 9.741, df = 3, p-value = 0.0209  **Goldfeld-Quandt test**  GQ = 0.41248, df1 = 8, df2 = 8, p-value = 0.8841  group value residals cooks leverage  1 Ctrl 8.056 -3.4410000 0.0106486030 0.1666667  2 Ctrl 9.219 -2.2780000 0.0046669240 0.1666667  3 Ctrl 15.762 4.2650000 0.0163591737 0.1666667  4 Ctrl 8.480 -3.0170000 0.0081860409 0.1666667  5 Ctrl 12.004 0.5070000 0.0002311741 0.1666667  6 Ctrl 15.461 3.9640000 0.0141315755 0.1666667  7 MP 38.990 7.4205000 0.0495210256 0.1666667  8 MP 44.700 13.1305000 0.1550550158 0.1666667  9 MP 39.826 8.2565000 0.0613077336 0.1666667  10 MP 36.079 4.5095000 0.0182885837 0.1666667  11 MP 17.497 -14.0725000 0.1781007737 0.1666667  12 MP 12.325 -19.2445000 0.3330707963 0.1666667  13 NP 25.575 2.6881667 0.0064988375 0.1666667  14 NP 32.044 9.1571667 0.0754128804 0.1666667  15 NP 22.377 -0.5098333 0.0002337651 0.1666667  16 NP 22.407 -0.4798333 0.0002070638 0.1666667  17 NP 17.916 -4.9708333 0.0222219259 0.1666667  18 NP 17.002 -5.8848333 0.0311452360 0.1666667  19 Mix 30.565 -0.7401667 0.0004926997 0.1666667  20 Mix 21.682 -9.6231667 0.0832835649 0.1666667  21 Mix 29.544 -1.7611667 0.0027894861 0.1666667  22 Mix 42.180 10.8748333 0.1063575956 0.1666667  23 Mix 28.506 -2.7991667 0.0070466193 0.1666667  24 Mix 35.354 4.0488333 0.0147429058 0.1666667  **One-way analysis of means (not assuming equal variances)**  F = 16.023, num df = 3.000, denom df = 10.352, p-value = 0.0003267  **Games_howell_test**  # A tibble: 6 × 8  .y. group1 group2 estimate conf.low conf.high p.adj p.adj.signif  * *<chr>* *<chr>* *<chr>* *<dbl>* *<dbl>* *<dbl>* *<dbl>* *<chr>*  1 valor Ctrl MP 20.1 0.296 39.8 0.047 *  2 valor Ctrl NP 11.4 3.00 19.8 0.01 **  3 valor Ctrl Mix 19.8 9.50 30.1 0.001 ***  4 valor MP NP -8.68 -28.4 11.0 0.498 ns  5 valor MP Mix -0.264 -20.2 19.6 1 ns  6 valor NP Mix 8.42 -2.71 19.5 0.157 ns  *- CAT (micromoles of H₂O₂ degraded × min⁻¹ × mg protein⁻¹)*   \| Ctrl \| MP \| NP \| Mix \| \| --- \| --- \| --- \| --- \| \| 0.039 \| 0.05 \| 0.05 \| 0.039 \| \| 0.049 \| 0.032 \| 0.043 \| 0.068 \| \| 0.043 \| 0.065 \| 0.05 \| 0.054 \| \| 0.044 \| 0.056 \| 0.051 \| 0.065 \| \| 0.041 \| 0.043 \| 0.042 \| 0.056 \| \| 0.045 \| 0.044 \| 0.048 \| 0.05 \|   **Mean ± Std. Deviation**  **Ctrl** = 0.044 ± 0.003  **MP** = 0.048 ± 0.011  **NP** = 0.047 ± 0.004  **Mi**x = 0.055 ± 0.011  **Dixon test for outliers**  **Ctrl -** Q = 0.4, p-value = 0.3651  **MP** - Q = 0.27273, p-value = 0.7567  **NP**- Q = 0.11111, p-value = 0.5533  **Mix** - Q = 0.37931, p-value = 0.4177  **Shapiro-Wilk normality test**  W = 0.95293, p-value = 0.3132  **Levene's Test for Homogeneity of Variance (center = mean)**    Df F value Pr(>F)  group 3 2.65 0.07673 .  20  **Modified robust Brown-Forsythe Levene-type test based on the**  **absolute deviations from the median**  Test Statistic = 2.5358, p-value = 0.08577  **Fligner-Killeen test of homogeneity of variances**  Fligner-Killeen:med chi-squared = 6.0835, df = 3, p-value = 0.1076  **studentized Breusch-Pagan test**  BP = 6.2191, df = 3, p-value = 0.1014  **Goldfeld-Quandt test**  GQ = 0.87912, df1 = 8, df2 = 8, p-value = 0.5701  group value resiuals cooks leverage  1 Ctrl 0.039 -0.0045000000 0.0181410974 0.1666667  2 Ctrl 0.049 0.0055000000 0.0270996641 0.1666667  3 Ctrl 0.043 -0.0005000000 0.0002239642 0.1666667  4 Ctrl 0.044 0.0005000000 0.0002239642 0.1666667  5 Ctrl 0.041 -0.0025000000 0.0055991041 0.1666667  6 Ctrl 0.045 0.0015000000 0.0020156775 0.1666667  7 MP 0.050 0.0016666667 0.0024884907 0.1666667  8 MP 0.032 -0.0163333333 0.2389946497 0.1666667  9 MP 0.065 0.0166666667 0.2488490730 0.1666667  10 MP 0.056 0.0076666667 0.0526564639 0.1666667  11 MP 0.043 -0.0053333333 0.0254821451 0.1666667  12 MP 0.044 -0.0043333333 0.0168221973 0.1666667  13 NP 0.050 0.0026666667 0.0063705363 0.1666667  14 NP 0.043 -0.0043333333 0.0168221973 0.1666667  15 NP 0.050 0.0026666667 0.0063705363 0.1666667  16 NP 0.051 0.0036666667 0.0120442951 0.1666667  17 NP 0.042 -0.0053333333 0.0254821451 0.1666667  18 NP 0.048 0.0006666667 0.0003981585 0.1666667  19 Mix 0.039 -0.0163333333 0.2389946497 0.1666667  20 Mix 0.068 0.0126666667 0.1437352246 0.1666667  21 Mix 0.054 -0.0013333333 0.0015926341 0.1666667  22 Mix 0.065 0.0096666667 0.0837128282 0.1666667  23 Mix 0.056 0.0006666667 0.0003981585 0.1666667  24 Mix 0.050 -0.0053333333 0.0254821451 0.1666667  **One-way analysis of means**  F = 2.1805, num df = 3, denom df = 20, p-value = 0.122  - GLUCOSE (µmol mg protein^-1^)   \| Ctrl \| MP \| NP \| Mix \| \| --- \| --- \| --- \| --- \| \| 11.391358 \| 9.408443 \| 13.777382 \| 9.802913 \| \| 12.926831 \| 9.867445 \| 10.074583 \| 9.922766 \| \| 12.835897 \| 9.231102 \| 10.286402 \| 9.787756 \| \| 11.070897 \| 9.332010 \| 14.471244 \| 9.245010 \| \| 12.911232 \| 8.666153 \| 10.797293 \| 17.414405 \| \| 12.600101 \| 7.866673 \| 6.683858 \| 16.618842 \|   **Mean ± Std. Deviation**  group variable n mean sd  *<fct>* *<fct>* *<dbl>* *<dbl>* *<dbl>*  1 Ctrl value 6 12.3 0.834  2 MP value 6 9.06 0.701  3 NP value 6 11.0 2.82  4 Mix value 6 12.1 3.80  **Dixon test for outliers**  **Ctrl -** Q = 0.17267, p-value = 0.8389  **MP** - Q = 0.39959, p-value = 0.3656  **NP**- Q = 0.43541, p-value = 0.2857  **Mix** - Q = 0.097383, p-value = 0.4881  **Shapiro-Wilk normality test**  W = 0.94523, p-value = 0.2131  **Levene's Test for Homogeneity of Variance (center = mean)**    Df F value Pr(>F)  group 3 8.2349 0.000915 ***  20  **Modified robust Brown-Forsythe Levene-type test based on the**  **absolute deviations from the median**  Test Statistic = 1.4478, p-value = 0.2588  **Fligner-Killeen test of homogeneity of variances**  Fligner-Killeen:med chi-squared = 3.0053, df = 3, p-value = 0.3908  **studentized Breusch-Pagan test**  BP = 10.128, df = 3, p-value = 0.01751  **Goldfeld-Quandt test**  GQ = 18.865, df1 = 8, df2 = 8, p-value = 0.0001987  alternative hypothesis: variance increases from segment 1 to 2  group value residuals cooks leverage  1 Ctrl 11.391358 -0.8980280 0.0082079010 0.1666667  2 Ctrl 12.926831 0.6374453 0.0041355976 0.1666667  3 Ctrl 12.835897 0.5465110 0.0030398359 0.1666667  4 Ctrl 11.070897 -1.2184891 0.0151110848 0.1666667  5 Ctrl 12.911232 0.6218457 0.0039356606 0.1666667  6 Ctrl 12.600101 0.3107150 0.0009826003 0.1666667  7 MP 9.408443 0.3464724 0.0012217705 0.1666667  8 MP 9.231102 0.1691310 0.0002911379 0.1666667  9 MP 9.867445 0.8054739 0.0066032133 0.1666667  10 MP 9.332010 0.2700393 0.0007421749 0.1666667  11 MP 8.666153 -0.3958182 0.0015945713 0.1666667  12 MP 7.866673 -1.1952984 0.0145413597 0.1666667  13 NP 13.777382 2.7622549 0.0776568680 0.1666667  14 NP 10.074583 -0.9405438 0.0090034796 0.1666667  15 NP 10.286402 -0.7287249 0.0054047997 0.1666667  16 NP 14.471244 3.4561167 0.1215707707 0.1666667  17 NP 10.797293 -0.2178336 0.0004829499 0.1666667  18 NP 6.683858 -4.3312693 0.1909337706 0.1666667  19 Mix 9.802913 -2.3290361 0.0552083550 0.1666667  20 Mix 9.922766 -2.2091826 0.0496724537 0.1666667  21 Mix 9.787756 -2.3441925 0.0559292405 0.1666667  22 Mix 9.245010 -2.8869383 0.0848256836 0.1666667  23 Mix 17.414405 5.2824565 0.2840038544 0.1666667  24 Mix 16.618842 4.4868930 0.2049008666 0.1666667  **One-way analysis of means (not assuming equal variances)**  F = 15.947, num df = 3.00, denom df = 10.15, p-value = 0.0003629  **Games_howell_test**  # A tibble: 6 × 8  .y. group1 group2 estimate conf.low conf.high p.adj p.adj.signif  * *<chr>* *<chr>* *<chr>* *<dbl>* *<dbl>* *<dbl>* *<dbl>* *<chr>*  1 valor Ctrl MP -3.23 -4.60 -1.86 0.000156 ***  2 valor Ctrl NP -1.27 -5.46 2.91 0.724 ns  3 valor Ctrl Mix -0.157 -5.82 5.51 1 ns  4 valor MP NP 1.95 -2.25 6.15 0.427 ns  5 valor MP Mix 3.07 -2.61 8.75 0.313 ns  6 valor NP Mix 1.12 -4.88 7.12 0.936 ns \| \| --- \| --- \| --- \| --- \| --- \| --- \| --- \| --- \| --- \| --- \| --- \| --- \| --- \| --- \| --- \| --- \| --- \| --- \| --- \| --- \| --- \| --- \| --- \| --- \| --- \| --- \| --- \| --- \| --- \| --- \| --- \| --- \| --- \| --- \| --- \| --- \| --- \| --- \| --- \| --- \| --- \| --- \| --- \| --- \| --- \| --- \| --- \| --- \| --- \| --- \| --- \| --- \| --- \| --- \| --- \| --- \| --- \| --- \| --- \| --- \| --- \| --- \| --- \| --- \| --- \| --- \| --- \| --- \| --- \| --- \| --- \| --- \| --- \| --- \| --- \| --- \| --- \| --- \| --- \| --- \| --- \| --- \| --- \| --- \| --- \| |

**PCoA_PERMANOVA:**

Permutation test for adonis under reduced model

Terms added sequentially (first to last)

Permutation: free

Number of permutations: 10000

adonis2(formula = vars.scaled ~ Group, data = dados, permutations = 10000, method = "euclidean")

Df SumOfSqs R2 F Pr(>F)

Group 3 68.372 0.42467 4.9209 9.999e-05 ***

Residual 20 92.628 0.57533

Total 23 161.000 1.00000

---

Signif. codes: 0 ‘***’ 0.001 ‘**’ 0.01 ‘*’ 0.05 ‘.’ 0.1 ‘ ’ 1

*****VECTORS**

Dim1 Dim2 r2 Pr(>r)

EROD -0.99972 -0.02379 0.1053 0.3030

GST -0.98963 0.14365 0.6271 9.999e-05 ***

SOD 0.28054 0.95984 0.8062 9.999e-05 ***

CAT -0.60345 0.79740 0.5119 0.0002 ***

TBARS -0.29804 0.95455 0.7610 9.999e-05 ***

PCO -0.99622 -0.08683 0.6772 0.0002 ***

Glucose -0.83169 -0.55524 0.6132 0.0002 ***

---

Signif. codes: 0 ‘***’ 0.001 ‘**’ 0.01 ‘*’ 0.05 ‘.’ 0.1 ‘ ’ 1

Permutation: free

Number of permutations: 10000
